# Information normally considered task-irrelevant drives decision-making and affects premotor circuit recruitment

**DOI:** 10.1038/s41467-022-29807-2

**Published:** 2022-04-19

**Authors:** Drew C. Schreiner, Christian Cazares, Rafael Renteria, Christina M. Gremel

**Affiliations:** 1 Department of Psychology, University of California San Diego, La Jolla, CA 92093 USA; 2 The Neurosciences Graduate Program, University of California San Diego, La Jolla, CA 92093 USA

**Keywords:** Operant learning, Cortex, Decision

## Abstract

Decision-making is a continuous and dynamic process with prior experience reflected in and used by the brain to guide adaptive behavior. However, most neurobiological studies constrain behavior and/or analyses to task-related variables, not accounting for the continuous internal and temporal space in which they occur. We show mice rely on information learned through recent and longer-term experience beyond just prior actions and reward - including checking behavior and the passage of time - to guide self-initiated, self-paced, and self-generated actions. These experiences are represented in secondary motor cortex (M2) activity and its projections into dorsal medial striatum (DMS). M2 integrates this information to bias strategy-level decision-making, and DMS projections reflect specific aspects of this recent experience to guide actions. This suggests diverse aspects of experience drive decision-making and its neural representation, and shows premotor corticostriatal circuits are crucial for using selective aspects of experiential information to guide adaptive behavior.

## Abstract (web summary)

Prior experience is used by the brain to guide adaptive behaviour during decision making. Here, the authors show that mice also selectively use information learned through recent and longer-term experience beyond just prior actions and reward to guide adaptive behaviour.

## Introduction

Investigations into the neuroscience of decision-making are typically aimed at understanding how ongoing brain computations support behavior. As decision-making is a continuous and dynamic process, one’s past and ongoing experience is likely to be reflected in and used by the brain to guide decision-making. While we may exploit learned rules and associations, our ongoing experiences (related-and-unrelated to such rules and associations) can shape our behavior. That experience modulates decision-making is broadly evident and has been the focus of significant study; it is found in innate behaviors^1^, and though experience can drive initial learning, simple win-stay and lose-shift experience-based strategies persist even after rules and associations are well-learned. Indeed, experience continues to influence behavior even when detrimental to task performance (e.g., in many perceptual tasks)^2,3^. That the use of experience to guide decision-making is often altered in psychiatric disease (e.g., the temporal pattern of drug use, and not just total amount of drug, is decisive in substance use disorders^4^), suggests efforts towards investigating its role in guiding decision-making are warranted.

However, investigations into how experience shapes decision-making and recruits neural mechanisms are often impoverished. Historically, tasks investigating decision-making often institute a trial structure, limit choice and movement, and elicit behavior via cues, with the latter resulting in a focus on elicited stimulus-response characterization of involved mechanisms^5^. More recently, there has been a growing focus on the contribution of prior choices, actions, and outcomes^2,6–8^ as sources of experiential information. However, by examining these contributions based on their discrete occurrence (e.g., a binary choice), this experiential information has been removed from the rich, continuous environment in which it is naturally embedded and across which it evolves. Thus, the potential contribution of seemingly task-irrelevant behaviors is also typically neglected. There is growing concern that such an approach negates the individualistic and continuous nature of decision-making^9–13^. Presumably, continuous experiential information is used by the brain to execute adaptive behavior to support ongoing decision-making. Yet such information is often treated as task-irrelevant and may be ignored or factored out^14^.

This neglect may result in seemingly incongruent hypotheses when decision-making and its neural mechanisms are likely to rely on such experiences. One example may be hypotheses concerning secondary motor cortex (M2) and its role in exploration versus experience-guided behaviors. On one hand, M2’s sensory, motor, and premotor characteristics have suggested a role in using experience to guide decision-making^7,15–17^. On the other hand, several studies implicate M2 in implementing stochastic or exploratory decisions^18–21^. However, animals may decide to explore based upon their experience; for instance making more exploratory decisions when uncertainty is high^22^. Thus, attribution of M2 function to seemingly disparate processes may reflect the lack of accounting for, or limiting the contribution of, prior experiences.

Here, we hypothesize that M2 represents and integrates continuous experiential information to guide experience or exploration-based decision-making when use of such information is advantageous. We utilize an unstructured free operant foraging task with continuous analog variables in mice where experience provides the only information available to guide performance. We show aspects of experiential information normally considered task-irrelevant, such as the passage of time and checking behaviors, play large roles in supporting and adjusting adaptive behavior. We then show M2 circuits are key for broad aspects of experiential control while selective M2 output to dorsal medial striatum (DMS) conveys action-related aspects of experience to drive adaptive behavior.

## Results

### Mice learned a self-generated, self-paced lever press hold down task

**Fig. 1:**
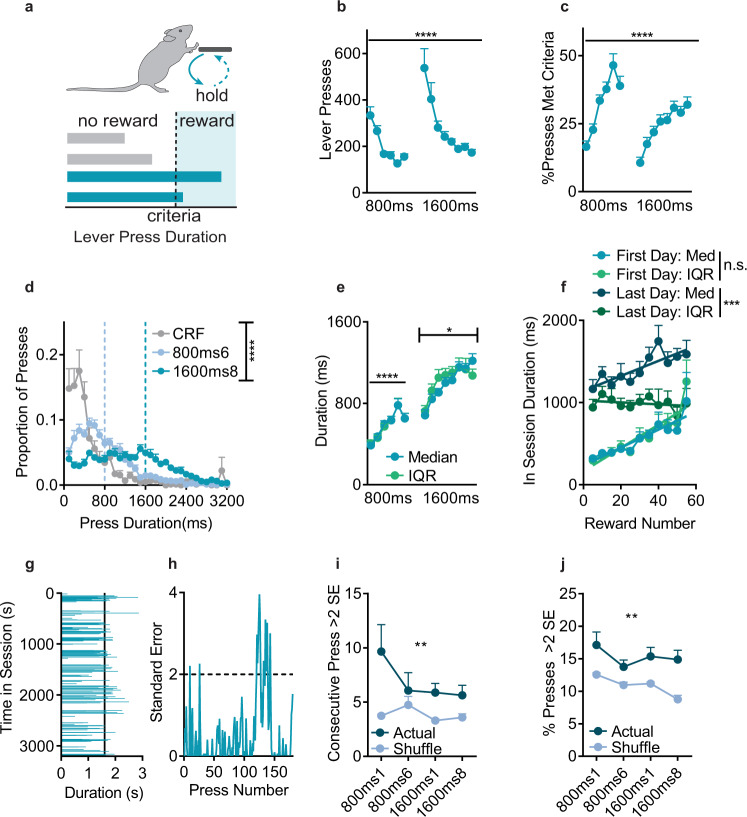
Mice learned an unstructured, self-generated, self-paced lever press hold down task. **a** Behavioral schematic; mice learn to press and hold down a lever for at least a minimum duration to earn food reward. **b** Total Lever Presses across training days (1-way ANOVA, main effect of day, F_2.9, 31.9_ = 12.0, *p* < 0.0001). **c** %Presses met criteria (1-way ANOVA, main effect of day F_4.22, 46.5_ = 17.2, *p* < 0.0001). **d** Histogram of lever press durations (100 ms bins) on the final pretraining day (CRF = Continuous Ratio of Reinforcement), and final 800 ms and 1600 ms days. Dashed lines indicate criterion. 2-way RM ANOVA, main effect of Duration Bin F_31,1056_ = 34.1, *p* < 0.0001, and an interaction (Duration Bin/Criterion) F_62,1056_ = 10.5, *p* < 0.0001. **e** Median and Interquartile Range (IQR) of lever press durations (800 ms training: 2-way RM ANOVA, main effect only of Day, F_5,55_ = 19.5, *p* < 0.0001. 1600 ms training: 2-way RM ANOVA, main effect of Day F_7,77_ = 14.0, *p* < 0.0001, and interaction (Median/IQR x Day) F_7,77_ = 2.44, *p* = 0.026). **f** Duration median (Med) and IQR within a session, grouped by cumulative rewards. Linear regressions found non-zero slopes for Med on the first (F_1,110_ = 28.9, *p* < 0.0001, R^2^ = 0.21) and final (F_1,115_ = 12.6, *p* = 0.0006, R^2^ = 0.099) training day, while IQR had a non-zero slope on the first (F_1,110_ = 48.5, *p* < 0.0001, R^2^ = 0.306) but not last (F_1,115_ = 0.28, *p* = 0.59, R^2^ = 0.002) day. Med/IQR slopes did not differ on the first (F_1,220_ = 1.2, *p* = 0.027), but did differ by the final day (F_1,230_ = 9.1, *p* = 0.003). **g** Sample behavior of one trained mouse showing press durations in order of occurrence. **h** Upper cumulative sum from the same mouse/session. **i**, **j** Number of consecutive presses (**i**) and Overall % of presses (**j**) that were >2 Standard Errors (SE) above the mean in the upper cumulative sum. 2-way RM ANOVA, difference from order shuffled data for % (F_1,11_ = 17.1, *p* = 0.0017) and number of consecutive presses (F_1,11_ = 14.0, *p* = 0.0032). First days excluded, F’s_1,11_ > = 4.94, p’s < 0.05. All tests were two-tailed and corrected for multiple comparisons. 800 ms and 1600 ms refer to days where criterion was >800 ms or >1600 ms. ****p < 0.0001, ***p < 0.001, **p < 0.01, *p < 0.05. *n* = 12 mice. Points represent mean + SEM across mice, unless noted otherwise. See also Supplementary Fig. 1, Source Data.

We adapted an instrumental task^23–25^ where mice (*n* = 12 C57BL/6 J) were trained to press and hold down a lever for at least a minimum duration to earn a food reward, with reward delivered after lever press release/offset (Fig. 1a). There were no external cues signaling reward availability or duration, nor any trial structure (lever was always available). Thus lever presses were self-initiated, self-paced, and self-terminated and mice had to explore to determine the rule, a process termed action differentiation^26^.

We first examined macroscopic aspects of lever pressing. Mice were initially trained with a >800 ms criterion before being shifted to a > 1600 ms criterion. Mice readily learned that press duration was the operant and quickly reduced the number of Total Lever Presses (Fig. 1b), while they increased the percentage of presses that met the minimum duration criterion (%Presses Met Criteria, Fig. 1c), and showed little evidence of stereotypies in their lever pressing (see Supplementary Note 1). Mice were sensitive to the minimum duration rule and shifted the distribution of press durations from a pretraining session with no duration requirement, to the final day of >800 ms training, and further still to the final day of >1600 ms training (Fig. 1d). To examine whether actions were controlled by their expected consequence and operationally goal-directed or were instead habitual^27,28^, we performed outcome devaluation testing (Supplementary Fig. 1a). Mice reduced their Total Lever Presses on Devalued days relative to Valued days (Supplementary Fig. 1b), consistent with using expected outcome value to guide decisions as seen in goal-directed control^27^. Although Total Lever Presses decreased, the %Presses Met Criteria increased following devaluation (Supplementary Fig. 1c) with a small rightward shift in the distribution of press durations (Supplementary Fig. 1d), suggesting action selection and execution may be differentially controlled by outcome value.

It is clear that mice can use contingency and consequence information to perform this task, but it is unclear how they are doing so. One possibility is that executed lever press durations are independently timed. If so, we hypothesized that mice may exhibit the scalar property of timing; as lever press durations increase, so too does variability^25,29^. During initial short criterion training, we found concomitant increases in both the median duration and the interquartile range (IQR) of each animal’s lever press across training days (Fig. 1e). However, when duration criteria increased, the pattern of change in lever press IQR departed from that of median duration. Mice showed within session increases in median durations across training (Fig. 1f), but increases in IQR were present on the first, but not the last training day. Furthermore, while the within session median and IQR slopes were not different on the first day, they did differ by the final day. Finally, the IQR/Median ratio significantly changed between the final day of 800 ms versus 1600ms training (Wilcoxon signed rank test, W_11_ = 78.0, *p* = 0.0005), in violation of scalar timing.

Violation of the scalar property could emerge as a result of mice using experiential or non-timing information rather than simply timing presses independently. In Fig. 1g, for one well-trained mouse, representative press durations plotted across a session show variability in when presses occurred and in their duration, as well as seemingly distinct periods of reduced variability. A cumulative sum (upper bound) analysis (Fig. 1h) uncovered prolonged periods of time when mice emitted press durations >2 standard errors above the mean, (Fig. 1i, j). This was not due to random chance, the consequence of very long press durations inflating the cumulative sum, or an artifact of early learning. Overall, this suggests that there was local patterning of lever pressing within individuals.

### Varied experiences shape continuous decision-making

**Fig. 2:**
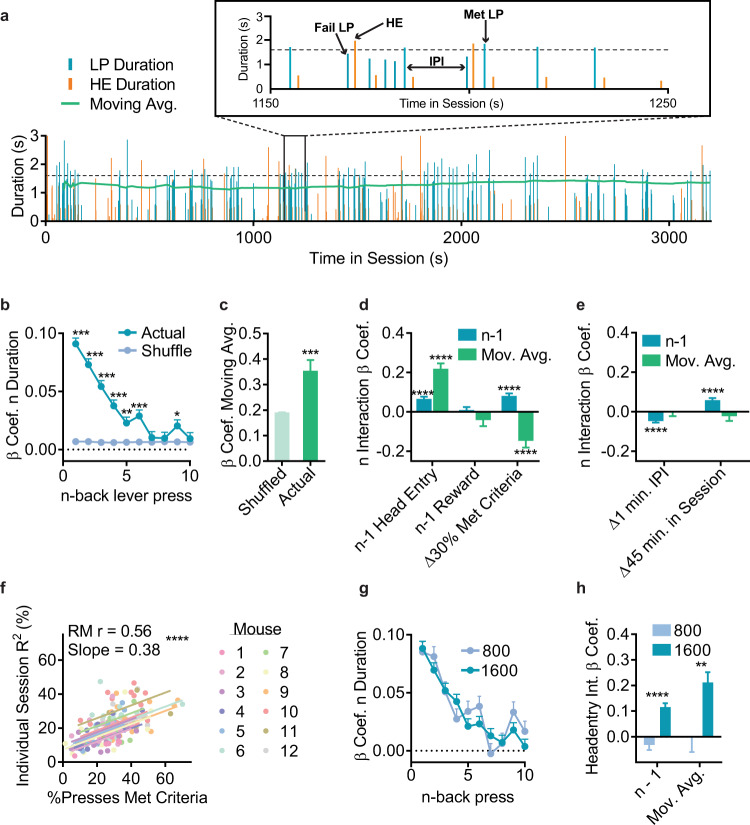
Experience contributes to self-generated decision-making. **a** Sample data from one mouse (as in Fig. 1g, h) showing diversity of experiential information available. Top shows a zoomed in subset. Dashed line indicates 1600 ms criterion. **b** LME model β coefficients relating current lever press duration (*n*) to preceding press durations (*n* - x) for Actual and order Shuffled data. **c** Moving average β coefficient for Actual and Shuffled data. **d**, **e** β coefficients for the interaction between experiential variables and recent (*n* - 1 duration) or long-term (moving average) experience. For display purposes we transformed continuous variables to show relevant changes, e.g. time in session, which is in units of ms, was transformed to 45 min (half the duration of a session). **f** Repeated measures correlation between task performance (%Presses Met Criteria) and model fit (R^2^) for linear models built using individual session data. Intercept was allowed to vary across mice, while keeping a shared slope (Rm = 0.56, DF = 153, *p* < 0.0001, slope = 0.38). **g** As in b only building LME models using data only from 800 ms training (800) or 1600 ms training (1600). 2-way RM ANOVA (800/1600 x n-back), main effect only of n-back F_9,399440_ = 27.1, *p* < 0.0001. **h** As in (**d**) and (**e**), building complex LME models using only either 800 or 1600 data. Only HE terms significantly differed between 800/1600 models (unpaired *t* tests with false discovery correction, HE*n - 1: t_40232_ = 5.90, *p* < 0.0001, HE*Mov. Avg.: t_40232_ = 3.05, *p* = 0.0023). All tests were two-tailed and corrected for multiple comparisons. Coef = β coefficient. Int = Interaction. LP = Lever Press, HE = Headentry into food magazine, IPI = Inter Press Interval. Mov. Avg. = Moving Average. Δ = Change. *Markers in (**b**, **c**) indicate comparisons to order shuffled data, (**d**, **e**) indicate significant F-tests on model terms. ****p < 0.0001, ***p < 0.001, **p < 0.01, *p < 0.05. Data points are mean + SEM. Shuffled data are mean + SEM of 1000 order shuffled β coefficients. See also Table 1, Supplementary Fig. 2, Supplementary Tables 1, 2, and Source Data.

The relative similarity among serial lever presses suggests that durations from recently executed lever presses may contribute. We created a simple linear mixed effect model (LME) to predict current press duration (*n*) given recently executed durations (n-back). We included random effects of both training day and mouse to account for the repeated structure of our data. We also included several control variables and compared the actual coefficients to those obtained from order shuffled data using permutation tests (Supplementary Table 1). We found a consistent significant linear relationship between current press *n* duration and the durations of *n* - 1 through *n* - 6 presses, with the magnitude of this relationship decaying across n-back presses (Fig. 2b) with largely similar *n* - 1 coefficients across mice (Supplementary Fig. 2a). Importantly, there was a positive correlation with a mouse’s *n* - 1 duration coefficient and overall performance (Supplementary Fig. 2b), suggesting mice that used recent experience to a larger degree were able to perform more efficiently.

However, recent lever presses are not the only experiential information available (Fig. 2a). The unstructured nature of this self-generated task allows us to capture aspects of decision-making that occur across a continuous space beyond just press duration, including reward delivery, checking behavior, and the inter-press interval between press *n* and press *n* - 1. We created more complex LMEs, first building a “full” model that included *n* - 1 through *n* - 6 durations, as well as main effect and interaction terms for other n-back variables (see Table 1 for terms). We performed backwards selection on this full model using Bayesian Information Criterion, meaning that the addition of these variables helped to explain additional variance in press durations (Methods). Permutation tests found these variables differed from order shuffled data (shuffled within a single mouse/session). Similar to the simple LME model, mice that had more similar adjacent presses had more efficient performance (Supplementary Table 2 30% increase in %Met Criteria roughly doubles the *n*/*n* - 1 relationship). Importantly, the history of executed durations beyond the recently executed lever presses contributed, as evidenced by a significant moving average coefficient (Fig. 2c; permutation test *p* < 0.001).

**Table 1:** Complex LME Model Parameters, related to Fig. 2.

Model Parameters	Description
Dur_n-1_… Dur_n-6_	Durations of prior lever presses from *n* - 1 to *n* - 6.
MA	A moving average of lever press durations from *n* - 7 up to *n* - 60.
HE_n-1_	A binary variable coding for if a checking headentry (HE) was (1) or was not (0) made in between lever press *n* and press *n* - 1.
Rew_n-1_	A binary variable coding for if lever press *n* - 1 was (1) or was not (0) rewarded.
IPI_n-1_	Inter press interval (IPI, in ms) between lever press *n* and press *n* - 1.
IPI_n-2_	As above, only for the IPI between press *n* and press *n* - 2.
Time	A timestamp (in ms) for when in a session a lever press occurred (from 0 ms to 5,400,000 ms).
%Met	Overall % of presses that met criteria for a given session
Dur_n-1_: Interactions	Interaction terms between *n* - 1 Duration and: *n* - 1 Headentry, *n* - 1 Reward, *n* - 1 IPI, Time in Session, and %Met Criteria. Overall question is whether the use of recent durations (*n* - 1) is affected by these variables.
MA: Interactions	Interaction terms between the Moving Average (MA) and: *n* - 1 Headentry, *n* -1 Reward, *n* - 1 IPI, Time in Session, and %Met Criteria. Overall question is whether the use of the long-term moving average is affected by these variables.

However, mice have additional experiences beyond just pressing the lever. As reward delivery can serve as a feedback signal to adjust behavior^30,31^, we examined whether its presence (or absence) altered the relationship between sequential lever presses. While there was a small but significant negative main effect of reward delivery (suggesting mice made shorter presses after reward, perhaps to titrate press durations near the criterion boundary, as has been previously suggested^24^), surprisingly, reward delivery did not alter the *n* - 1 coefficient (Fig. 2d). One potential explanation is that reward-related information may already be captured within the learned contingency (duration) itself, as presses above a certain duration are rewarded. To more directly investigate this, we imposed a probabilistic reward schedule in a separate cohort (25%, 50%, or 75% rewarded, *n* = 5 mice/group) following training. While imposing a probabilistic schedule increased efficient performance (Supplementary Fig. 2c), a lever press that met criteria (Met) led to an increased relationship between *n* and *n* - 1, whether that press was rewarded or not. Indeed, the magnitude of this effect was larger when the Met press was unrewarded due to the probabilistic schedule, with this “win-stay” effect more pronounced in the 25% probability group. This provides additional evidence mice use an internal representation of which press durations are related to reward to guide future durations and rely less on the presence (or absence) of reward delivery.

Freely moving subjects also have the opportunity to gain information about success likelihood through checking behaviors, such as head-entries (HE) into the food receptacle (Supplementary Table 2). Indeed, mice used information from checking behavior to adjust lever press durations (Fig. 2d). The magnitude of this increase was quite large: lever presses within a lever press/HE/lever press sequence were effectively twice as related to one another relative to those in a lever press/lever press sequence. The above findings challenge the assumption that decision-making is solely determined by the serial order of actions and their outcome, as is often presumed in trial-based experimental designs. That sources of this crucial experiential information accrue across a continuous temporal space raises the question of how the passage of time itself may influence decision-making. The relationship between two adjacent presses (presses *n* and *n* - 1) decreased as the inter-press-interval (IPI) increased (Fig. 2e). To give an example of the magnitude, the model predicts that the relationship between *n* and *n* - 1 would be ~0 if they were separated by 120 s. This raises the hypothesis that animals may rely more on the long-term moving average to guide their behavior following long IPIs^32^. Indeed, the use of long-term experience was unaffected by the IPI. Further, we found that *n* and *n* - 1 became more similar towards the end of a session, and again, there was no relationship between time in session and the moving average (Fig. 2e).

Collectively, these results suggest that individual experiences, including checking behavior and the passage of time, are crucial for modifying recent experience to guide behavior. This is evident in the strong positive correlation between task performance and model R^2^ when ran on individual mouse/session data (Fig. 2f). We built separate LMEs using only 800 ms or 1600 ms data to examine how the use of experience itself evolved across learning. While mice used prior duration information even early in learning (Fig. 2g), the ability to use headentry checking information emerged with additional training (Fig. 2h). Although our goal was not to make the most accurate predictions, the final model did predict 24.1% of all press durations within a 95% CI, and whether a press did/did not meet criteria 73.8% of the time. Thus, in the final model (Supplementary Table 2), the use of experience correlated with performance.

### M2 represents prior experience to guide exploration

**Fig. 3:**
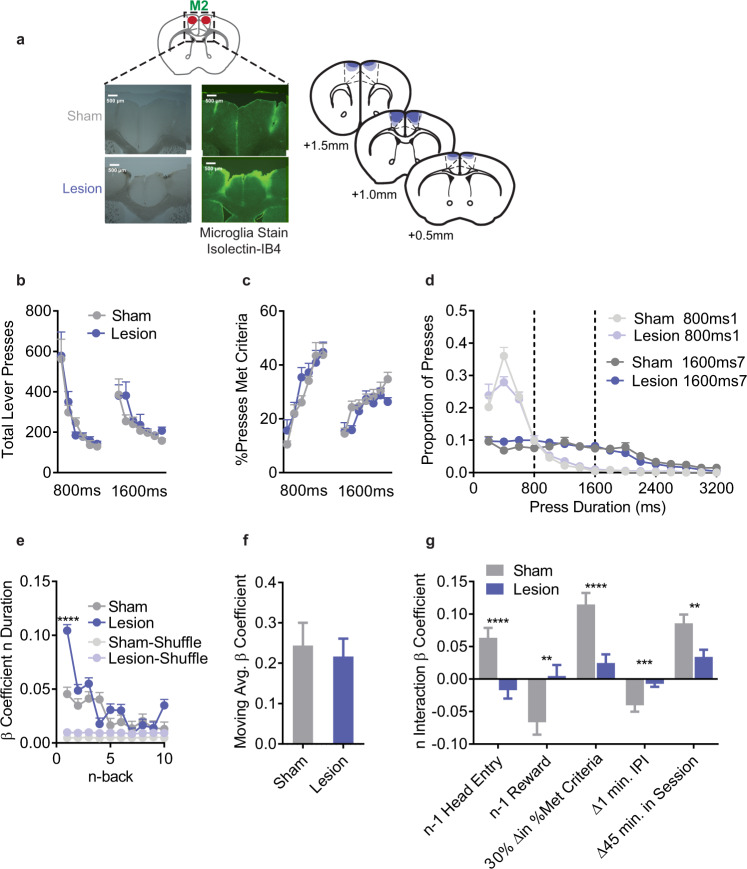
Pretraining lesions of M2 impair integration of experiential information. **a** (top left) Schematic, (bottom left) sample histology, and (right) average/maximal (dark/light shading) spread of excitotoxic lesions of M2, slice coordinates relative to Bregma. **b** Total Lever Presses across training days. **c** %Presses that met criteria across training days. **d** Histogram of lever press durations on the first and last days of the 800 ms and 1600 ms criteria (200 ms bins). **e** LME model β coefficients predicting *n* duration from n-back durations for Actual and order Shuffled data. 2-way ANOVA (n-back x Sham/Lesion) main effect of n-back (F_9,572740_ = 20.2, *p* < 0.0001) and Sham/Lesion (F_1,572740_ = 14.1, *p* = 0.0002) and significant interaction (F_9,572740_ = 6.33, *p* < 0.0001). **f** β coefficient for the moving average term. **g** β coefficients for the interaction terms from the complex LME model. For display purposes we transformed the continuous variables to show relevant changes. All tests were two-tailed and corrected for multiple comparisons. **b**–**d** are mean+SEM across mice. Shuffled data are the mean + SEM of 1000 order shuffled β coefficients. ****p < 0.0001, ***p < 0.001, **p < 0.01. See also Supplementary Table 3 for statistical comparisons, Source Data.

M2 has been reported to be involved in both exploration and experience-based decision-making, and this apparent discrepancy may be due to neglecting the contribution of seemingly task-irrelevant variables. Therefore, we performed pretraining lesions of M2 using ibotenic acid (Fig. 3a; Lesion *n* = 10) or vehicle (Sham *n* = 8). In line with prior reports^25^, we found no differences between Sham and Lesion mice in coarse behavioral measurements such as Total Lever Presses (Fig. 3b), %Presses Met Criteria (Fig. 3c), or Press Durations (Fig. 3d). However, M2 lesioned mice executed lever press durations that were more similar to their prior action (Fig. 3e). Post-hoc testing revealed a significant group difference only at *n* - 1 (t_572740_ = 6.87, *p* < 0.0001 (Fig. 3f). This was confirmed in a joint LME built with both Sham and Lesion mice together, which also revealed a significant Sham/Lesion group interaction with *n* - 1 duration (coefficient for the Lesion group relative to the Sham = +0.047, F_1,57271_ = 37.6, *p* = 8.77e–10). Using the complex LME model, M2 lesions abolished all *n* - 1 duration interactions with Reward, Checking, IPI, and Time in Session (Fig. 3g, Supplementary Table 3). Lesions did not affect moving average interactions. Collectively, this suggests M2 lesioned mice were relatively inflexible, and were left to rely on the just-made action without integration of broad experiential information.

**Fig. 4:**
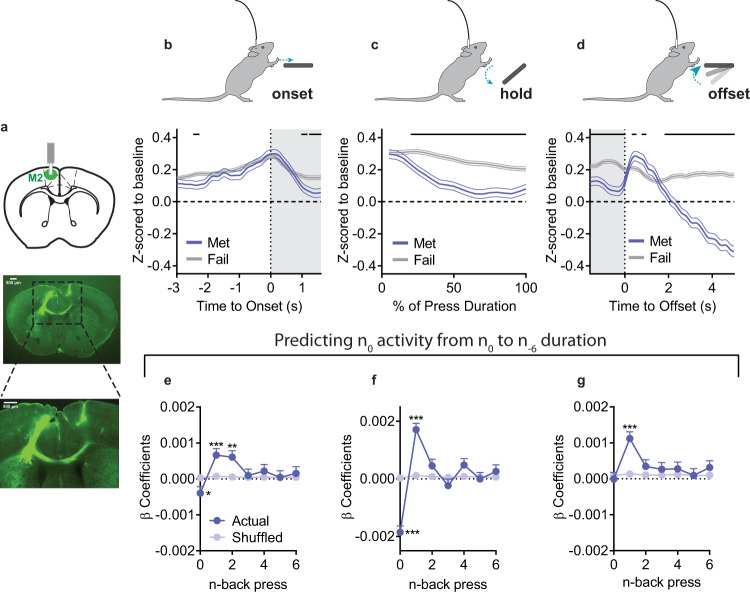
M2 Ca^2+^ activity reflects prior experience. **a** (top) Schematic and (bottom) example histology of M2 in vivo Ca^2+^ fiber photometry recordings. **b**–**d** Ca^2+^ activity z-scored relative to a baseline period and aligned to (**b**) press onset, (**c**) the hold down period itself (presented as the relative % of a press’s duration), and (**d**) the offset of a lever press. **e**–**g** β coefficients from LME models relating activity to current and prior durations for Actual and order Shuffled data (**e**) before press onset, (**f**) during the press, and (**g**) after press offset. Met = Presses that met criterion, Fail = Presses that did not meet criterion. Grey shading in (**b**, **d**) indicates 1.6 s. Black lines in (**b**–**d**) indicate significant differences between Met/Fail via permutation testing that required consecutive samples to pass the threshold for significance. All tests were two-tailed, and **e**–**g** were corrected for multiple comparisons. Shuffled data are the mean + SEM of 1000 order shuffled β coefficients. ***p < 0.001, **p < 0.01, *p < 0.05. See also Supplementary Fig. 3, Supplementary Table 4, and Source Data.

How may experience influence representation of decision-making in M2 circuits? We utilized in vivo fiber photometry (Fig. 4a), and measured population Ca^2+^ activity from M2 excitatory neurons (*n* = 8). Aligning baseline z-scored activity to lever press onset (Fig. 4b), we observed preceding ramping activity as has been previously reported^16^. This ramping activity did not differ based on whether that press would go on to exceed the criteria duration (Met) or not (Fail) (permutation testing requiring 4 adjacent samples to pass the threshold for significance^33^). However, M2 activity during the lever press and at press offset was modulated by whether that lever press would or would not meet the criteria (Fig. 4c, d), with an abrupt increase in Ca^2+^ activity just after the offset of Met presses, – i.e. reward delivery – followed by a sustained reduction in activity (Fig. 4d). Analysis of mouse average data yielded largely similar patterns of activity (Supplementary Fig. 3). Thus, M2 activity is modulated during lever pressing with ongoing modulation reflecting future success.

To determine if M2 activity is related to ongoing and prior behavior, we created LME models to predict Ca^2+^ activity during epochs of the current lever press given both the ongoing action (press *n* duration) and prior behavior (the duration of press *n* - 1 to *n* - 6). We included prior activity as a covariate to control for autocorrelation in Ca^2+^ activity and compared β coefficients to 1000 order shuffled datasets. Before the onset of press *n*, there was a significant positive relationship between M2 activity and the just prior press durations (Fig. 4e; *n* - 1, *p* < 0.001; *n* - 2, *p* = 0.001) and a small negative relationship between activity and the upcoming duration (press *n*, *p* = 0.048). This representation of both current and prior lever press duration in M2 Ca^2+^ activity continued during the press itself (Fig. 4f; press *n*, *p* < 0.001; *n* - 1: *p* < 0.001). At press offset, there was no relationship with the just completed press (*n*), but there was a positive relationship with the previous lever press duration (Fig. 4g; *n* - 1, *p* < 0.001). This relationship was largely conserved across individual mice (LME models where relationship between durations and activity can vary by mouse; only 1–2 mice differed across any timepoint). Modeling with the complex LMEs showed M2 activity reflected: (main effects) Checking, IPI, and Time in Session, and their interactions with prior durations (Supplementary Table 4). In particular, HE checking behavior increased the relationship between lever press duration and M2 activity across lever press epochs. The complex LMEs also better predicted M2 Ca^2+^ activity relative to the simple LMEs that only included durations (difference in simple/complex prediction %: Before Press: +13.7%, During Press: +9.2%, After Press: +16.4%). Combined with lesion data, this suggests M2 circuits are recruited when a broad array of experiential information is used to guide behavior but not when behavior is accomplished using less flexible processes.

### M2-DMS projections reflect recent experience used to plan actions

**Fig. 5:**
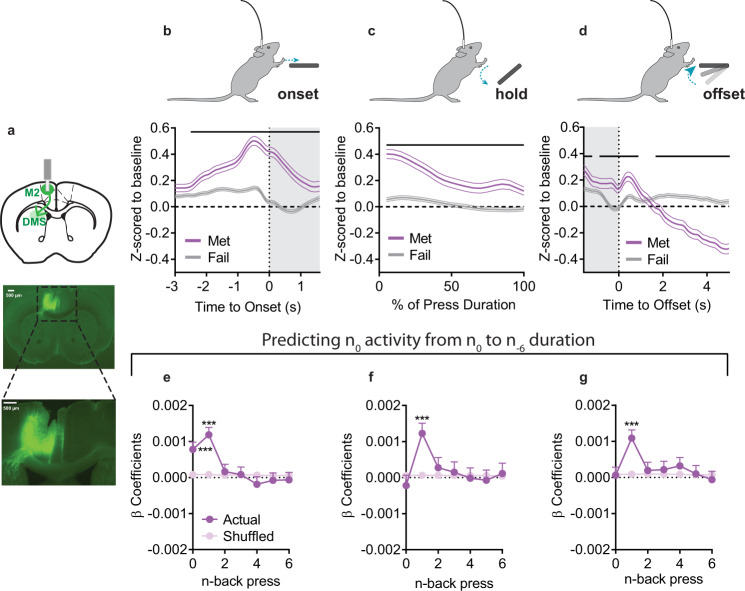
M2-DMS Ca^2+^ activity encodes preceding and upcoming actions. **a** (top) Schematic and (bottom) example histology of projection specific M2-DMS Ca^2+^ fiber photometry. **b**–**d** Ca^2+^ activity z-scored relative to baseline and aligned to (**b**) press onset, (**c**) the duration of the press, and (**d**) press offset. **e**–**g** β coefficients from LME models relating activity to current and prior durations for Actual and Shuffled data (**e**) before press onset, (**f**) during the press, and (**g**) after press offset. Met = Presses that met criteria. Fail = Presses that did not meet criterion. Grey shading in (**b**, **d**) indicates 1.6 s. Black lines in (**b**–**d**) indicate significant differences between Met/Fail via permutation testing that required consecutive samples to pass the threshold for significance. All tests were two-tailed, and (**e**–**g**) were corrected for multiple comparisons. Shuffled data are the mean + SEM of 1000 order shuffled β coefficients. ***p < 0.001. See also Supplementary Fig. 4, Supplementary Table 5, and Source Data.

M2 sends dense projections into dorsal medial striatum regions (M2-DMS)^34,35^ that contribute to action selection^36^, but it is unclear what information is conveyed to basal ganglia circuits. We performed in vivo fiber photometry of virally targeted M2-DMS activity and examined representation of experiential information within this population (*n* = 7, Fig. 5a). We again observed a ramping in M2-DMS Ca^2+^ activity prior to press onset. This activity reflected future success, with larger increases in activity for presses that would meet criteria (Fig. 5b). This relationship was also present during the press itself (Fig. 5c), upon lever release (Fig. 5d), and was replicated when using mouse average analyses of the Ca2+ signal (Supplementary Fig. 4), raising the hypothesis that M2-DMS projections may carry information involved in specifying and/or planning actions based on prior experience.

Indeed, LMEs using durations to predict M2-DMS activity before press onset showed both prior (Fig. 5e; *n* - 1, *p* < 0.001) and upcoming (*n*, *p* < 0.001) durations were positively related to M2-DMS Ca^2+^ activity. M2-DMS activity during the press did not relate to the current duration, but was positively related to the prior duration (Fig. 5f; *n* - 1, *p* < 0.001), and likewise at press offset there was a positive relationship only with *n* - 1 press duration (Fig. 5g; *p* < 0.001). As in M2, this was largely consistent across mice (on average, 1–2 mice had coefficients that differed from the overall coefficient at any timepoint). Furthermore, complex LMEs revealed significant main effects of Checking, prior Reward, IPI, and Time in Session on M2-DMS activity (Supplementary Table 5) and predicted more of the data relative to the simple models (difference in simple/complex prediction%: Before Press: +13.2%, During Press: +9.2%, After Press: +15.2%). Unlike M2, M2-DMS activity did not reflect modulatory interactions of Reward, IPI, or Time on *n* - 1 durations. Instead M2-DMS activity only reflected an interaction between *n* - 1 duration and checking, and this was observable across all lever press epochs (Supplementary Table 5).

**Fig. 6:**
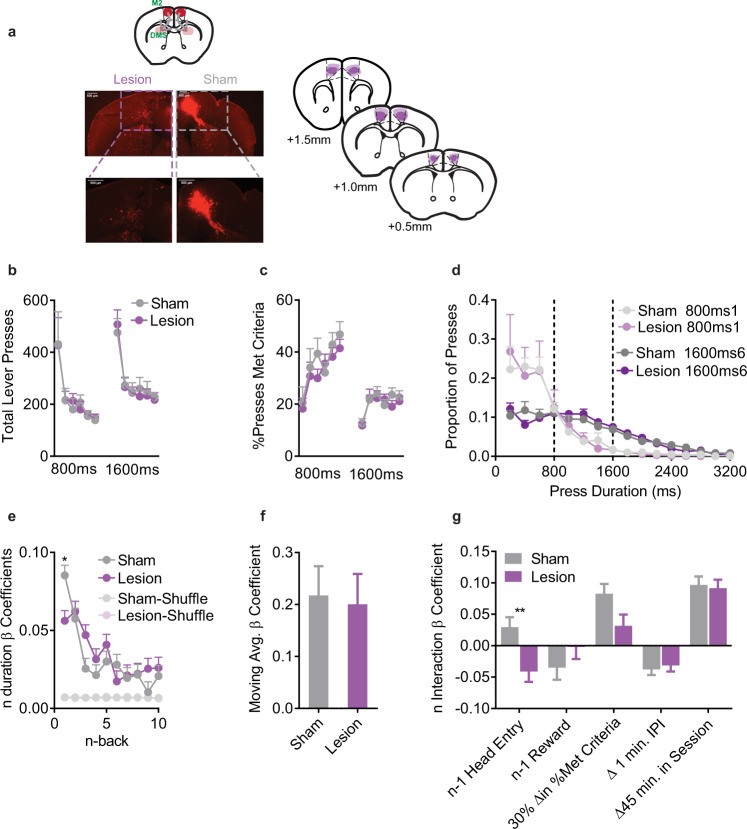
Pretraining M2-DMS lesions impair use of recent experience. **a** (top) Schematic, (bottom) example histology and (right) average/maximal (dark/light shading) spread of projection specific M2-DMS lesion using a Cre-dependent caspase strategy, slice coordinates relative to Bregma. **b** Total Lever Presses across training days. **c** %Presses that met criteria across training days. **d** Histogram of lever press durations on the first and last days of the 800 ms and 1600ms criteria (200 ms bins). **e** β coefficient from LME models predicting n duration from n-back durations for Actual and order Shuffled data. 2-way ANOVA (n-back x Sham/Lesion) main effect of n-back (F_9,467760_ = 14.6, *p* < 0.0001); significant interaction (F_9,467760_ = 2.29, *p* = 0.0144). **f** Moving average β coefficient for Actual and Shuffled data. **g** β coefficients for the interaction terms from the complex LME model. For display purposes we transformed the continuous variables to show relevant changes. All tests were two-tailed and multiple comparisons corrected. **b**–**d** are mean + SEM across mice. Shuffled data are the mean + SEM of 1000 order shuffled β coefficients. **p < 0.01, *p < 0.05. See also Supplementary Table 6, Source Data.

To test whether M2-DMS activity functionally contributed to planning actions based on recent experience, we used a Cre-dependent caspase strategy to selectively lesion M2-DMS projection neurons prior to training (Fig. 6a, *n* = 8 Lesion, *n* = 8 Sham). Again, we observed no effect on coarse measures of behavior including Total Lever Presses, %Presses Met Criteria, and Press Durations (Fig. 6b–d). Simple LME modeling showed M2-DMS lesions reduced the relationship between press *n* and *n* - 1. This deficit was selective to *n* - 1 (multiple comparison corrected post-hoc *n* - 1: t_467760_ = 3.09, *p* = 0.021), and was replicated in an LME model built using both Sham and Lesion mice together (coefficient for the Lesion group relative to the Sham = −0.02, F_1, 46767_ = 5.60, *p* = 0.0179). There was no effect on the Moving Average (Fig. 6f). Interestingly, M2-DMS lesions produced a more specific deficit relative to the broad M2 lesions, as revealed by complex LMEs analyses. M2-DMS lesions reversed the contribution of a checking HE between press *n* and press *n* - 1, such that emitting another intervening behavior, checking, was detrimental to using prior duration information to guide performance (Fig. 6g; t_47352_ = 3.10, *p* = 0.002). No other terms differed between Sham and Lesion groups (Supplementary Table 6).

**Fig. 7:**
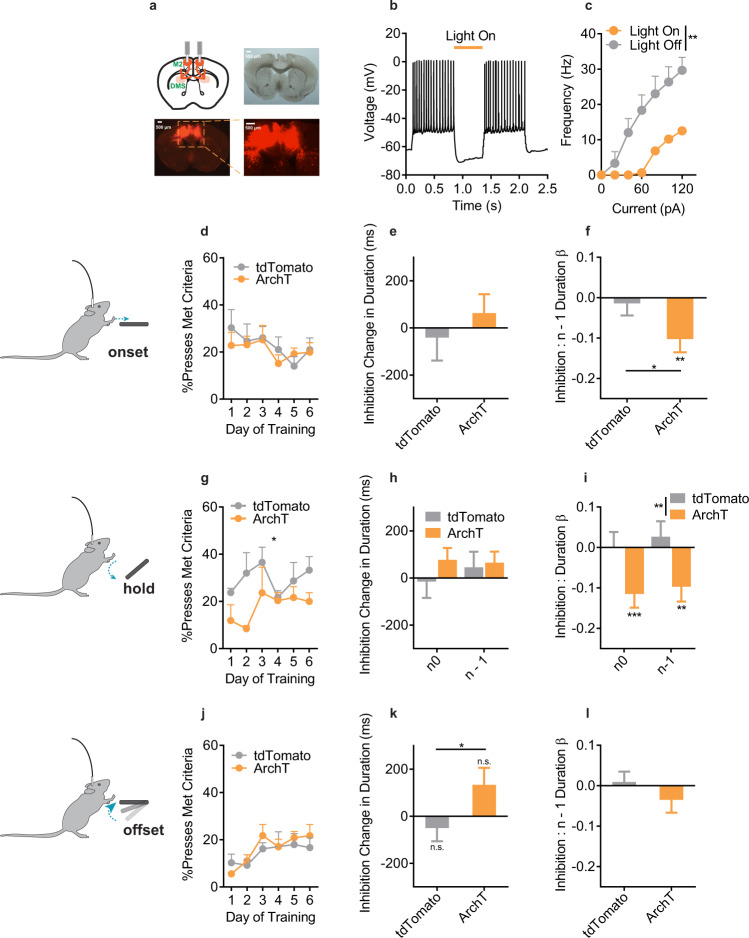
Optogenetic inhibition of M2-DMS projections during execution impairs use of recent experience. **a** (top left) Schematic and example histology of ArchT optogenetic inhibition of M2-DMS projection neurons. **b**, **c** Slice verification of ArchT-mediated inhibition in M2-DMS projection neurons. ** = 2-way RM ANOVA (Current x Light) interaction: F_6,6_ = 17.0, *p* = 0.002. **d**–**f** Pre-onset inhibition. **d** %Presses that met criteria, (**e**) Main effect of inhibition on duration, and (**f**) Interaction between inhibition and the contribution of the prior duration. **g**–**i** As in (**d**–**f**) except for inhibition during the duration of the press. In (**g**), a 2-way RM-ANOVA (Opsin/Fluorophore X Day) revealed a main effect only of Opsin group (F_1,9_ = 7.59, p = 0.0223), and in (**i**), a 2-way RM ANOVA (Opsin/Fluorophore x n-back) revealed a main effect only of Opsin group (F_1,23602_ = 10.5, *p* = 0.0012). **j**–**l** As in (**d**–**f**) except for inhibition occurring after press offset. Note that although there is a group difference between ArchT and tdTomato mice in (**k**), the main effect itself is n.s. in the model for both groups. All tests were two-tailed and multiple comparisons corrected. n0 = Light occurred on press n. n - 1 = Light occurred on press n - 1. tdTomato = tdTomato expressing control mice. ArchT = ArchT expressing mice. In all LME graphs, * without any lines indicate significant terms via F-test on the model (within group comparison), while * with a line indicate significant, between group differences. ***p < 0.001, **p < 0.01, *p < 0.05. n.s. = Not significant. Data are mean + SEM. See also, Source Data.

The photometry and lesion data together suggest that M2-DMS activity represents and is functionally necessary for recent sequential action experience (pressing and checking) to contribute to the initiation and execution of the current decision. To directly test this hypothesis, we took a behaviorally-dependent, closed-loop optogenetic approach to inhibit M2-DMS neural activity. We used a dual-virus strategy to express an inhibitory opsin (Fig. 7a, ArchT: *n* = 5) that reduced M2-DMS spiking when activated by light (Fig. 7b). We targeted inhibition to M2-DMS soma at three different epochs: the initiation of a lever press, during the press itself, and after lever press release. Each manipulation occurred across 6 days of training and only on a subset of lever presses. This allowed us to include additional terms in our LME models to determine if inhibition directly affected press *n* duration, and/or if inhibition affected the contribution of prior experience (i.e., an interaction between inhibition and *n* - 1 duration). In addition to this within subject comparison, we also made between subject comparisons to fluorophore control mice (tdTomato: *n* = 6).

To target inhibition prior to press onset, mice were tracked using an overhead camera and light (1 s, continuous) was triggered 50% of the time when mice entered a zone centered on the lever. We did not find any effect of pre-onset M2-DMS inhibition on overall performance (Fig. 7d), nor any effect on press duration itself (i.e., no main effect of inhibition, Fig. 7e). Within the ArchT group, inhibition prior to lever pressing did induce a significant negative interaction with *n* - 1 duration (F_1,2718_ = 10.6, *p* = 0.001), and a significant difference with the tdTomato group (Fig. 7f; t_6645_ = 2.04, *p* = 0.042). However, as this inhibition continued for 1 s, it may have persisted during lever pressing itself. Indeed, in analyses restricted to presses without any spillover, pre-onset inhibition had no effect even within the ArchT group (F_1,534_ = 0.45, *p* = 0.50)

We next tied inhibition to lever pressing itself. On every 7th lever press we inhibited during the full duration of the lever press, leaving M2-DMS activity intact during all other lever presses. Such inhibition did reduce the efficacy of lever pressing (Fig. 7g). Again, there was no main effect of inhibition on press duration itself, and this was true both whether inhibition occurred during press *n* or on press *n* - 1 (Fig. 7h). However, M2-DMS inhibition during press *n* prevented the use of *n* - 1 duration information from guiding the current action. Further, inhibition during press *n* also prevented the experiential information gained during the execution of that press from informing the next press (F-test within ArchT model, *n*: F_1,6110_ = 11.2, *p* = 0.0008; *n* - 1: F_1,6110_ = 6.91, *p* = 0.009), with effects significantly different than the tdTomato group (Fig. 7i). This suggests that M2-DMS activity during the press itself is not important for controlling the duration of the current lever press per se. Instead, this activity contributes to using recent experiential information to execute current actions, and this abrupt disruption impaired task performance. In support of this, when we targeted inhibition to press offset (1 s of light after press release), there was no direct effect of inhibition on subsequent press durations, nor an interaction with the use of recent experience, nor any effect on performance (Fig. 7j–l). There was also no interaction with the moving average term at any inhibition time point. The lack of a direct effect of inhibition on press duration suggests the deficit in use of recent experience was not due to a non-specific motor effect. The lack of any inhibition effect prior to or after execution of the lever press also suggests M2-DMS activity does not represent a form of working memory, but instead supports use of prior experience to inform current action execution.

## Discussion

There is a growing concern that neuroscience investigations into decision-making are “missing the forest for the trees”, or vice versa. Investigations into the nature of decision-making that isolate specific task-based computations or focus on summary statistics such as accuracy have been indispensable in providing information about both the tree and forest, respectively. However, the present data suggests the need to account for the mesoscopic context experiential information provides in order to link these levels of analysis, akin to understanding the intertwined communication among trees in a forest^37^. Here, sources of experiential information often treated as task-irrelevant determined whether and to what degree recent experience-information influenced behavior and recruited involved neural circuits. By using this approach, we show that M2 and M2-DMS circuits use broad experiential information to instigate exploratory or recent experience-based responding.

Classic temporal difference or reinforcement learning models^30,31^ emphasize the role played by responses and outcomes. By using an unconstrained task with a continuous decision variable, we found mice do not drastically shift their strategy solely on their sequence of actions, nor based on whether their action earned a reward. Rather, experiential variables such as checking and the passage of time strongly influenced behavior and may serve to arbitrate between strategies. Behaviorally, sensitivity to time could function to bias exploratory responding when the preceding action is more distal in time and hence, when the environment, and/or its neural representation may have changed. On the other hand, checking sensitivity suggests information seeking itself increases use of experience-based strategies, perhaps as a result of providing definitive feedback. As multiple behavioral controllers can be used to make seemingly similar decisions^38^, experiential information may be used to bias strategy-level recruitment, e.g., by adjusting the relative degree of exploration (Fig. 3) or the relative similarity between adjacent decisions (Figs. 6 and 7). This bias in recruitment strategy may arise through experiential modulation of associated neural activity^19^, perhaps by setting the “gain” on behavioral strategies^39^.

We found a robust representation of varied experience-related information in broader M2 population activity. M2 lesions both increased the similarity between adjacent presses and reduced the integration of non-action-outcome information (including Checking, IPI, and Time). M2 might receive such information from associative regions such as orbital frontal cortex^19^, as well as sensory regions^40^. Although the M2 Sham comparison group showed a low *n* - 1 β coefficient, the increased *n* -1 duration relationship is still present when comparisons are made to another sham group (M2-DMS Shams; t_56426_ = 2.22, *p* = 0.026), and there was a similar loss of interactions with other experiential information. Collectively, our results extend previous studies implicating M2 populations in goal-directed or model-based decision-making^41^ by providing insight into precisely how this effect is achieved. Namely, by nullifying the potential for an individual’s recent experience to contribute to decision-strategy arbitration, animals with M2 lesions rely on repetition-based strategies.

While M2 is important for a broader representation of experiential information, we saw a subset of M2 projections neurons (M2-DMS) provided a more limited representation of information. Converging Ca^2+^ activity, lesion, and optogenetic inhibition studies implicate M2-DMS projections specifically in contributing recent lever press and checking information to ongoing actions. The reduction in the use of recent experience only when optogenetic inhibition occurred during the press suggests that M2-DMS activity may serve as an experience-based guide or reference for ongoing actions. This raises the hypothesis that M2-DMS may function as part of a comparator for template or pattern matching during action performance, analogous to the pattern matching seen in avian vocal learning, and hypothesized to be implemented in premotor regions^42^. In M2-DMS lesioned mice, an intermediate behavior of checking in between lever presses reduced the reliance of the current action on the prior, providing some evidence that M2-DMS function is necessary to link and/or compare recent action experience as has been suggested by work examining sequence learning and initiation^43^. Future studies investigating M2-DMS function at the single neuron level could reveal important insights into precisely how this is instantiated in the brain, and if there is an “embodied engram” of recent actions, or a comparator function in M2-DMS projection neurons.

Rarely is behavior in the natural world so neatly constrained as in many laboratory tasks; thus it seems likely that animals have adapted to use diverse sources of information to guide their behavior. The brain should therefore be sensitive to this information, yet several recent studies have demonstrated remarkably widespread coding of variables in the brain^44,45^. Perhaps this apparent distributed coding is the consequence of attributing relatively static measurements of behavior and human-derived constructs to large neural populations. That there is a wealth of information available to animals and many neural circuits to support decision-making, raises the hypothesis that specific aspects of experiential information may modulate neural function differentially depending on the circuit and the computation. Investigations into circuit, synaptic, and molecular mechanisms controlling how such experience modulates decision-making will likely be fruitful, akin to increased understanding of arousal modulation of sensory processing^46^. Repetitive decision-making is found across many disease states including substance use disorders and obsessive compulsive disorder. M2’s potential human homologues - the pre-supplementary/supplementary motor areas, have shown promise for the targeting of transcranial magnetic stimulation in disease treatment^47–49^. Here we establish that M2-DMS is involved in implementing repetitive/recent-experience-based decisions. This raises the hypothesis that M2-DMS dysfunction may lead to decisions that are inappropriately or excessively repetitive^50^. Incorporating experience into the examination of disease-induced brain function during decision-making may increase the likelihood of obtaining enduring findings relevant to the clinical treatment of disease.

## Methods

### Experimental model and subject details

Similar numbers of male and female C57BL/6 J mice (>7 weeks/50 PND) (The Jackson Laboratory, Bar Harbour, ME) were used for experiments. Exploratory analyses for sex differences in the behavioral cohort revealed no differences, and thus we collapsed across sex. All procedures were conducted during the light period and mice had free access to water throughout the experiment. Mice were housed 2–4 per cage on a 14:10 light:dark cycle, housed at 71.6 °F, at 30% humidity. Mice were at least 6 weeks of age prior to surgery. Mice were food restricted to 85–90% of their baseline weight 2 days prior to the start of behavioral procedures, and were fed 1–4 h after the daily training. All experiments were approved by the University of California San Diego Institutional Animal Care and Use Committee and were carried out in accordance with the National Institutes of Health (NIH) “Principles of Laboratory Care”.

### Behavioral procedures

Mice were trained once per day in operant chambers in sound attenuating boxes (Med-Associates, St Albans, VT) in which they pressed a lever (left or right of the food magazine, counterbalanced for location) for an outcome of regular ‘chow’ pellets (20 mg pellet per reinforcer, Bio-Serv formula F0071). Each training session commenced with an illumination of the house light and lever extension and ended after either 60 reinforcers were earned or 90 min had elapsed, with the lever retracting and the house light turning off.

On the first day, mice were trained to approach the food magazine to retrieve the pellet outcome (no lever present) on a random time (RT) schedule, with a reinforcer delivered on average every 120 s for a total of 60 min. Next, mice were trained on a continuous ratio schedule of reinforcement (CRF) across 3 days, where every lever press was reinforced (no duration requirement), with the total possible number of reinforcers increasing (CRF10, 30, and 60).

Following CRF pretraining, the hold down task was introduced. We instituted a duration requirement on lever pressing: animals had to press and hold down the lever for >800 ms in order to earn food reward (delivered immediately after press release). Importantly, there were no cues, no timeout period, nor any discrete trials; the lever was always available to mice, until they completed their session. Mice were trained at the >800 ms criterion for 6 days, followed by at least 6 days of training at the >1600 ms criterion. During all days, timestamps for lever press onset, lever press offset, the onset and offset of headentry into the food magazine, and the delivery of reward were recorded. From this timing information, we were able to calculate durations of lever presses and headentries. Of note, use of Med Associates introduced a 20 ms limit on our time resolution.

### Outcome devaluation

In the behavioral mice (Figs. 1, 2 and Supplementary Figs. 1, 2, *n* = 12 total, *n* = 7 female and *n* = 5 male), after 8 days of training at >1600 ms, we performed outcome specific satiety. Devaluation procedures occurred across two days. In brief, on the valued day, mice had *ad libitum* access to an outcome previously experienced in the home cage for 1 h before being placed in the training context for a 5 min, non-reinforced test session. On the devalued day, mice were given 1 h of ad libitum access to the outcome previously earned by lever press, and then underwent a 5 min, non-reinforced test session in the training context. One mouse consumed <0.1 g of the valued outcome during pretraining exposure and was excluded from all devaluation analyses (giving final *n* = 11). The order of revaluation day was counterbalanced across mice.

### Probabilistic reward

A naive group of mice (*n* = 15, *n* = 4 female and *n* = 11 male) were trained for 6 days on >800 ms, followed by 8 days at >1600 ms criteria, and then switched to probabilistic reward, where only a percentage of presses that exceeded the duration criterion were rewarded on a random ratio schedule. These animals were separated into three different probabilistic reward groups: 25%, 50%, and 75% (*n* = 5 each group) and trained for a further 3 days under the assigned probabilistic schedule.

### Surgical procedures

All viral vectors were obtained from the UNC Viral Vector Core (Chapel Hill, NC) or Addgene (Wateron, MA). Mice were anaesthetized with isoflurane (1–2%) and intracranial injections were performed via Hamilton syringe (Reno, NV) targeted at a relatively posterior portion of M2 (from Bregma: AP + 1.0 mm, L ± 0.5 mm and V − 1.2 mm, −1.4 mm from the skull), and/or DMS (from Bregma: AP + 1.0 mm, L ± 1.65 mm and V − 3.0 mm, −3.2 mm from the skull). Syringes were left in place for five minutes after each injection to allow for diffusion, and all viruses or drugs were infused at a rate of 100 nl/min. Mice were given at least two weeks to allow for recovery and viral expression before the start of experimental procedures (at least four weeks for all M2-DMS manipulations). After behavioral testing was concluded, mice were euthanized and brains were extracted and fixed in 4% paraformaldehyde. Localization and spread of viral expression was assessed in 50–100 µm thick brain slices using fluorescent microscopy (Olympus MVX10, Shinjuku, Japan).

For M2 lesions, *n* = 12 Lesion mice were bilaterally injected with ibotenic acid (10 mg/ml, ThermoFisher), while *n* = 12 Sham lesion mice were injected with vehicle (saline) at M2 (2 injections of 120 nl at V −1.4 mm and −1.2 mm from the skull in each hemisphere). In order to assess excitotoxic lesion presence and spread, brains were sliced at 50 um thick, and incubated with propidium iodide (1:10000 in 1xPBS, Chemodex: P0023) and Isolectin-GS IB_4_ Alexa Fluor 488 Conjugate (20:10000, ThermoFisher: l21411), a marker of microglial cells which are recruited via lesions^51^. Brain slices were incubated for 1 hr, followed by 3 x 10 min washes. 4 Sham mice were excluded due to technical difficulties during training, and 2 Lesion mice were excluded due to histology, giving final n’s of 10 Lesion (*n* = 6 female, *n* = 4 male) and 8 Sham (*n* = 4 female, *n* = 4 male) mice.

For M2 GCaMP experiments, *n* = 8 mice (*n* = 4 female, *n* = 4 male) were injected (2 injections of 200 nl at V −1.4 mm and 1.2 mm from the skull) with rAAVDJ/PAAV-CaMKIIa-GCaMP6s to express GCaMP6s under control of the Ca^2+^ calmodulin dependent protein kinase IIα (CamKIIα) promoter and implanted with an optical fiber unilaterally in M2.

For M2-DMS GCaMP experiments, *n* = 8 mice were unilaterally injected with a viral vector expressing Cre recombinase (AAV5/Ef1a-Cre-WPRE) in DMS (2 injection depths: V −3.0 mm and −2.8 mm from the skull, 250 nl each), and were injected with a viral vector expressing a Cre-dependent GCaMP6s (pAAV.CAG.FLEX.GCaMP6s.WPRE.SV40 (Addgene: 100842); 2 injection depths: V: −1.4 mm, and −1.2 mm from the skull, 200 nl each) followed by fiber implantation in ipsilateral M2. One mouse was excluded due to histology (final *n* = 4 female, *n* = 3 male).

For M2-DMS lesion, *n* = 8 Lesion (*n* = 4 female, *n* = 4 male) and *n* = 8 Sham (*n* = 4 female, *n* = 4 male) mice were bilaterally injected with 200 nl of a viral vector expressing CamKIIα-Cre in DMS (rAAV5/CamKII-GFP-Cre; 2 injection depths: V: −3.1 mm and −2.9 mm from skull, 200 nl each). Lesion and Sham mice were also injected with a viral vector expressing Cre-dependent tdTomato in M2 (rAAV5/Flex-tdTomato; 100 nl at V −1.4 mm from the skull). Lesion mice additionally received a viral vector expressing a Cre-dependent caspase virus in M2 to induce apoptosis of M2-DMS projections (rAAV5/AAV-Flex-taCasP3-TEVP; 2 injection depths: V −1.4 mm and −1.2 mm from the skull, 200 nl each).

For M2-DMS optogenetic inhibition experiments, *n* = 8 Archaerhodopsin (ArchT) and *n* = 8 tdTomato mice were bilaterally injected with a viral vector expressing CamKIIα-Cre in DMS (rAAV5/CamKII-GFP-Cre; 2 injection depths: V −3.1 mm and −2.9 mm from the skull, 200 nl each). Due to the proximity of bilateral M2 at this posterior portion (~1.0 mm) for ferrule implantation, we injected virus and implanted fibers at a 10° angle, and adjusted the M2 coordinates accordingly. Experimental ArchT mice received a viral vector expressing a Cre-dependent inhibitory opsin (rAAV5/Flex-ArchT-tdTomato), while fluorophore control mice received a viral vector expressing Cre-dependent fluorophore only (rAAV5/Flex-tdTomato), in both cases receiving the same injection volume (300 nl at V −1.42 mm from the skull), with bilateral M2 fibers implanted at V −1.37 mm from the skull. Following exclusion for viral expression or low levels of behavior, there were *n* = 5 ArchT mice (*n* = 3 male, *n* = 2 female), and *n* = 6 tdTomato control mice (*n* = 3 male, *n* = 3 female).

### Fiber photometry

Animals underwent pre-training as described above, but received one additional day of CRF training during which animals were first hooked up to 400 um optical fiber tethers with ferrule to ferrule connectivity. A 470 nm LED (Thorlabs, Newton, NJ) was used for excitation of GCaMP6s (<70 µW/mm2), and fluorescence emissions were collected with a bifurcated fiber (Thorlabs, Newton, NJ) which allowed for simultaneous, independent recordings of two mice. We imaged the dual fiber core using a 4x objective (Olympus, Shinjuku, Japan) focused onto a CMOS camera (FLIR Systems, Wilsonville, OR). Regions of interest (i.e., the fiber cores) were selected using Bonsai software^52^ to acquire fluorescence intensity signals (at a rate of 20 Hz). Bonsai software simultaneously collected analog behavioral data and timestamps for lever presses, head entries, and reinforcer delivery sent via TTL Med-PC pulses using microprocessors (Arduino Duo, from Arduino, Sumerville, MA) with custom code. Photometry and behavioral data were imported into Matlab 2019b (Mathworks Inc., Natick, MA) for analysis using custom scripts. To account for decay across the session (photobleaching), we fit the fluorescence intensity signal to a double exponential. To check for bad coupling of the fiber to the ferrule, or low expression, each session we calculated the 97.5 percentile of dF/F and ensured that there was at least a 1% change, sessions failing to meet this criterion were excluded from analyses^53^, and also excluded sessions with visual anomalies in the session long traces (e.g., a sudden, sustained decrease in activity partway through the session that could indicate fiber decoupling). We used the mean and standard deviation during a baseline period -15s to -5s prior to lever pressing to z-score press-related activity (i.e., from -5s prior to onset up to 5 s post offset). To compare Met and Fail lever presses, we performed running permutation tests, requiring that at least 4 adjacent samples were significantly different from one another to control for fluctuations in the data (functions implemented in Matlab from^33^). We smoothed Ca^2+^ activity data using a 10 sample (or 5 sample for interpolated activity) long Gaussian filter for display purposes only.

### Optogenetic inhibition

For bilateral light delivery, Arduino Duos with custom code were used to receive TTL pulses from Med-PC operant chambers and trigger onset of 2 LEDs (595 nm, Thorlabs) coupled to 200 um sheathed fiber optic cable with ferrule-to-ferrule connectivity (> = 1 mW at ferrule tip). We used 595 nm light as this has been shown to optimally activate ArchT while avoiding non-specific effects^54^. We used several different protocols to target the closed-loop inhibition to different task epochs. Inhibition during the duration of the lever press occurred across the 6 > 800 ms training days, with light delivery (continuous, not pulsed) tied to the lever pressing itself. As we observed a decaying relationship between n-back press durations and *n* - 0 press duration, every 7th lever press triggered light delivery, which persisted for the duration of the lever press (with a time resolution of 20 ms for light offset). During days 1–6 of the >1600 ms training, we instead tied light delivery to press offset, again, on every 7th lever press. Thus, after every 7th lever press, mice were given 1 s of light. Finally, after undergoing 4 days of baseline training without any light inhibition (though while still being hooked up to fibers), we shifted to inhibiting prior to press onset for 6 days. To achieve this, animals were recorded with an overhead camera (1080p wide angle webcam, Logitech) and tracked in real time using Bonsai software. We individually defined regions of interest centered on the lever (approximately twice the width and length of the lever itself). 50% of entrances into this region generated a TTL pulse to turn on the LEDs, which remained on for 1 s.

### ArchT slice validation

Coronal slices (250 μm thick) containing M2 were prepared using a Pelco easiSlicer (Ted Pella Inc, Redding, CA). Mice were anesthetized by inhalation of isoflurane and brains were rapidly removed and placed in 4 °C oxygenated ACSF containing the following (in mM): 210 sucrose, 26.2 NaHCO_3_, 1 NaH_2_PO_4_, 2.5 KCl, 11 dextrose, bubbled with 95% O_2_/5% CO_2_. Slices were transferred to an ACSF solution for incubation containing the following (in mM): 120 NaCl, 25 NaHCO_3_, 1.23 NaH_2_PO_4_, 3.3 KCl, 2.4 MgCl_2_, 1.8 CaCl_2_, 10 dextrose. Slices were continuously bubbled with 95% O_2_/5% CO_2_ at pH 7.4, 32 °C and were maintained in this solution for at least 60 min prior to recording.

Whole-cell current clamp recordings were made in pyramidal cells of M2. Pyramidal cells that expressed ArchT were identified by the fluorescent tdTomato label using an Olympus BX51WI microscope mounted on a vibration isolation table and a high-power LED (LED4D067, Thorlabs). Recordings were made in ACSF containing (in mM): 120 NaCl, 25 NaHCO_3_, 1.23 NaH_2_PO_4_, 3.3 KCl, 0.9 MgCl_2_, 2.0 CaCl_2_, and 10 dextrose, bubbled with 95% O_2_/5% CO_2_. ACSF was continuously perfused at a rate of 2.0 mL/min and maintained at a temperature of 32 °C. Picrotoxin (50 µM) was included in the recording ACSF to block GABAA receptor-mediated synaptic currents. Recording electrodes (thin-wall glass, WPI Instruments) were made using a PC-10 puller (Narishige International, Amityville, NY) to yield resistances between 3 and 6 MΩ. Electrodes were filled with (in mM): 135 KMeSO_4_, 12 NaCl, 0.5 EGTA, 10 HEPES, 2 Mg-ATP, 0.3 Tris-GTP, 260–270 mOsm (pH 7.3). Access resistance was monitored throughout the experiments. Cells in which access resistance varied >20% were not included in the analysis.

Recordings were made using a MultiClamp 700B amplifier (Molecular Devices, Union City, CA), filtered at 2 kHz, digitized at 10 kHz with Instrutech ITC-18 (HEKA Instruments, Bellmore, NY), and displayed and saved using AxographX (Axograph, Sydney, Australia). A series of fixed current injections (20 pA increments from 0 to 120 pA) were used to elicit action potential firing and the number of spikes were counted at each current step. For verification of ArchT function, ArchT was optically stimulated using 590 nm light, delivered via field illumination using a high-power LED (LED4D067, Thorlabs). Optical stimulation was done under constant illumination for 1 s during current injections.

### Data analysis

#### Linear mixed effects models of behavior

We built simple Linear Mixed Effects (LME) models to model the relationship between the duration of lever press *n* and n-back (*n* - 1 through *n* - 10) lever press durations. We included random intercept terms for mouse and day of training to account for the repeated structure of our data. To determine how far back a significant relationship existed between press *n* and any particular n-back press, we shuffled the order of a particular n-back (e.g., only *n* - 3) 1000 times and compared the shuffled distribution of beta coefficients to the actual value via permutation test. Of note, we are shuffling here within individual mouse/sessions, thus preserving the overall statistics of the data, and shuffling only the order in which a specific type of event occurred.

1 $$n={\beta }_{0}+{\beta }_{n-1}{n}_{-1}+{\beta }_{n-2}{n}_{-2}+\ldots +{\beta }_{n-10}{n}_{-10}+{\beta }_{t}(t)+{\beta }_{ \% }( \% )+(1|M)+(1|D)+{\varepsilon }_{i}$$

In Eq. 1, *n* is the current lever press duration, *n* - 1 through *n* - 10 are the previous 1 through 10 lever press durations and *β*_*x*_ is the linear regression coefficient for term *x* (*β*_*0*_ is the intercept). We also included covariates of time in session (*t*) and the percentage of presses that met criteria (*%*). We included random intercept terms for both mouse (*M*) and day (*D*).

In order to determine which other experiential variables affected lever press n duration, we also built more complex LME models that included additional variables. To select variables for this model, we created a “full” model that included n-back durations up to *n* - 6 (as that is as far back as we see a consistent difference from shuffled data in the simple models), and then main effects of other variables and their interactions with n-back durations, also up to *n* - 6 (e.g., a binary for if mice made a checking headentry after the previous lever press). We individually removed terms from this full model and compared Bayesian Information Criterion (BIC) to assess if adding a term improved the model. If any term did not improve the model, we removed it, and also removed any further n-back examples of it. However, we kept main effect terms in the model if the interactions were significant and kept all the same interaction terms for *n* - 1 and the moving average term to be able to directly compare how various events might differentially affect the contribution of press *n* - 1 versus the moving average. To ensure that terms in this reduced model did not improve the model due to overall correlations across days or mice, we also compared beta coefficients from the actual data to those obtained from 1000 order shuffled datasets, where we individually permuted a given term within individual mouse/sessions. This analysis conducted on our “reduced” model agreed with the BIC selection for terms that improved the model. We were ultimately left with the model in Supplementary Table 2 (see also Table 1 for a description of terms), signified by Eq. 2 below.

2 $$n= {\,\,}{\beta }_{0}	+{\beta }_{n-1}{n}_{-1}+{\beta }_{n-2}{n}_{-2}+\ldots +{\beta }_{n-6}{n}_{-6}+{\beta }_{{{{{{\rm{MA}}}}}}}{{{{{\rm{MA}}}}}}+{\beta }_{t}t\\ 	+{\beta }_{ \% } \% +{\beta }_{{{{{{\rm{IPI}}}}}}}{{{{{{\rm{IPI}}}}}}}_{-1}+{\beta }_{{{{{{\rm{IPI}}}}}}}{{{{{{\rm{IPI}}}}}}}_{-2}+{\beta }_{R}{R}_{-1}\\ 	+{\beta }_{{{{{{\rm{HE}}}}}}}{{{{{{\rm{HE}}}}}}}_{-1}+{\beta }_{t\ast n-1}t\ast n-1+{\beta }_{ \% \ast n-1} \% \ast n-1+{\beta }_{{{{{{\rm{IPI}}}}}}\ast n-1}{{{{{{\rm{IPI}}}}}}}_{-1}\ast n-1\\ 	+{\beta }_{{{{{{\rm{IPI}}}}}}\ast n-2}{{{{{{\rm{IPI}}}}}}}_{-2}\ast n-2+{\beta }_{R\ast n-1}{R}_{-1}\ast n-1+{\beta }_{{{{{{\rm{HE}}}}}}\ast n-1}{{{{{{\rm{HE}}}}}}}_{-1}\ast n-1\\ 	+{\beta }_{t\ast {{{{{\rm{MA}}}}}}}t\ast {{{{{\rm{MA}}}}}}+{\beta }_{ \% \ast {{{{{\rm{MA}}}}}}} \% \ast {{{{{\rm{MA}}}}}}+{\beta }_{{{{{{\rm{IPI}}}}}}\ast {{{{{\rm{MA}}}}}}}{{{{{{\rm{IPI}}}}}}}_{-1}\ast {{{{{\rm{MA}}}}}}\\ 	+{\beta }_{R\ast {{{{{\rm{MA}}}}}}}{R}_{-1}{{{{{\rm{MA}}}}}}+{\beta }_{{{{{{\rm{HE}}}}}}\ast {{{{{\rm{MA}}}}}}}{{{{{{\rm{HE}}}}}}}_{-1}\ast {{{{{\rm{MA}}}}}}+(1|M)+(1|D)+{\varepsilon }_{i}$$

Where *β*_*x*_ represents the linear regression coefficient for a given term. This model has the same terms as the simple model, though only back to *n* - 6 durations, as that is as far back as there is a reliable difference to shuffled data. In addition, there is the MA term which is a moving average from presses *n* - 7 through *n* - 60 (length selected via BIC using different window lengths). Additionally, we have main effects of time in session (*t*, in ms), the percentage of presses that met criteria (*%*), inter-press interval (IPI in ms, for both time in between press *n* and press *n* - 1 (IPI_-1_), and between press *n* and press *n* - 2 (IPI_-2_)), outcome of press *n* - 1 (*R*_*-1*_: binary where 0 is no reward and 1 is reward), and headentry between press *n* - 1 and press *n* (HE_-1_: binary where 0 is no headentry and 1 is headentry). Again, we have random intercept terms for mouse (*M*) and day of training (*D*). We also included interaction terms between the *n* - 1 duration term and: *t*, *%*, IPI, *R*_*n-1*_, and HE_n-1_. These interaction terms are specified with the general format of *β*_x*n-1_x*n-1 where *x* represents an individual interaction term (e.g., for time in session *t* interacting with *n* - 1 duration: *β*_t*n-1_t*n-1).These same interaction terms were included with the moving average term (MA, of the general format *β*_x*MA_x*MA) in order to see if very recent experience (*n* - 1) and long-term experience (MA) were differentially influenced by variables such as time. Interestingly, when examining further n-back interactions, only the interaction between IPI_n-2_ and *n* - 2 duration survived the BIC selection process, indicating that individual further n-backs were less open to modification by these variables.

In the probabilistic reward experiment, we added a trinary term for if a lever press was unsuccessful (0), successful and unrewarded (1), or successful and rewarded (2), and included interactions between this term and *n* - 1 as well as the MA. Additionally, we ran all three probability groups together in the model and included indicator variables for which group (25%, 50%, or 75% reward) a mouse belonged to. This allowed us to include a 3-way interaction to determine if the groups differed in how this trinary outcome term interacted with prior press durations (e.g., does the probability of reward influence the presence/magnitude of win-stay behavior?). For the optogenetic inhibition LME models, we included a binary term indicating if a lever press received light delivery (before, during, or after for the three different manipulations) as both a main effect and as an interaction with *n* - 1 duration and the MA to determine if light reduced the relationship between press *n* and press *n* - 1/the MA.

#### Ca^2+^ activity linear mixed effect models

For the M2 and M2-DMS Ca^2+^ activity recordings, we built LME models that sought to predict Ca^2+^ activity given behavior. For this, we used data only from the 1600ms training days. First, we built simple LME models that included only current (*n*) and prior (n-back, up to *n* - 6) durations to predict activity (calculated as area under the curve) at three different time points: −1 s to 0 s before press onset, during the lever press itself, and 0 s to +1 s after press release. For activity during the lever press itself, we used modified Akima interpolation, implemented using Matlab’s interp1 function to get presses of different durations on the same relative scale, and we excluded any lever presses with fewer than 2 samples which would preclude interpolation. We also included terms for prior activity (up to *n* - 6) to control for autocorrelation in the Ca^2+^ activity signal. We again compared beta coefficients from the actual data to 1000 order shuffled datasets for these simple models.

3 $$A={\beta }_{0}+{\beta }_{n}{n}_{0}+{\beta }_{n-1}{n}_{-1}+\ldots +{\beta }_{n-6}{n}_{-6}+{\beta }_{A-1}{A}_{-1}+\ldots +{\beta }_{A-6}{A}_{-6}+(1|M)+(1|D)+{\varepsilon }_{i}$$

In Eq. 3, *A* is current Ca^2+^ activity and *β*_*x*_ is the regression coefficient for term *x*. Of note, these models included *n* duration (*n*_*0*_) as a predictor (whereas this was what we predicted in the pure behavioral models). We predicted *A* given both current (*n*_*0*_) and prior (*n* - 1, up to *n* - 6) press durations, included prior Ca^2+^ activity (*A* - 1, up to *A* - 6) as a covariate, and included random intercepts of mouse (*M*) and training day (*D*).

Additionally, we built more complex LME models to predict Ca^2+^ activity data. For these, we used the complex behavioral model above for the predictors, as we were interested in seeing if these variables - which we know influence the behavior - are also represented in Ca^2+^ activity, and we also still included prior Ca^2+^ activity data to control for autocorrelation in the Ca^2+^ data. This took the form of the following Eq. 4, using the same variables as the preceding equations.

4 $$A= {\,\,}{\beta }_{0}	+{\beta }_{A-1}{A}_{-1}+\ldots +{\beta }_{A-6}{A}_{-6}+{\beta }_{n0}{n}_{0}+{\beta }_{n-1}{n}_{-1}+\ldots +{\beta }_{n-6}{n}_{-6}\\ 	+{\beta }_{{{{{{\rm{MA}}}}}}}{{{{{\rm{MA}}}}}}+{\beta }_{t}t+{\beta }_{ \% } \% +{\beta }_{{{{{{\rm{IPI}}}}}}}{{{{{{\rm{IPI}}}}}}}_{-1}+{\beta }_{{{{{{\rm{IPI}}}}}}}{{{{{{\rm{IPI}}}}}}}_{-2}+{\beta }_{R}{R}_{-1}+{\beta }_{{{{{{\rm{HE}}}}}}}{{{{{{\rm{HE}}}}}}}_{-1}\\ 	+{\beta }_{t\ast n-1}t\ast n-1+{\beta }_{ \% \ast n-1} \% \ast n-1+{\beta }_{{{{{{\rm{IPI}}}}}}\ast n-1}{{{{{{\rm{IPI}}}}}}}_{-1}\ast n-1+{\beta }_{{{{{{\rm{IPI}}}}}}\ast n-2}{{{{{{\rm{IPI}}}}}}}_{-2}\ast n-2\\ 	+{\beta }_{R\ast n-1}{R}_{-1}\ast n-1+{\beta }_{{{{{{\rm{HE}}}}}}\ast n-1}{{{{{{\rm{HE}}}}}}}_{-1}\ast n-1+{\beta }_{t\ast MA}t\ast {{{{{\rm{MA}}}}}}+{\beta }_{ \% \ast {{{{{\rm{MA}}}}}}} \% \ast {{{{{\rm{MA}}}}}}\\ 	+{\beta }_{{{{{{\rm{IPI}}}}}}\ast {{{{{\rm{MA}}}}}}}{{{{{{\rm{IPI}}}}}}}_{-1}\ast {{{{{\rm{MA}}}}}}+{\beta }_{R\ast {{{{{\rm{MA}}}}}}}{R}_{-1}{{{{{\rm{MA}}}}}}+{\beta }_{{{{{{\rm{HE}}}}}}\ast {{{{{\rm{MA}}}}}}}{{{{{{\rm{HE}}}}}}}_{-1}\ast {{{{{\rm{MA}}}}}}+(1|M)+1(1|D)+{\varepsilon }_{i}$$

When trying to predict activity after lever press offset, we also included a binary term for outcome on lever press *n* (*R*_*0*_ i.e., the lever press that was just completed with 0 being no reward and 1 being reward). We did not include this term at the other time points since it would introduce a “post diction” confound (i.e., including a term for the outcome of a press before the press even occurred). For the same reason, we did not include interactions with the *n*_*0*_ variable.

#### Quantification and statistical analysis

All analyses were two-tailed with α = 0.05 as a threshold for significance. For analyzing coarse behavioral measurements (e.g., Total Lever Presses) one-way or two-way RM ANOVAs were used, with Greenhouse-Geisser correction for one-way ANOVA and Bonferroni corrections for post-hoc multiple comparisons unless otherwise noted. We used the RMcorr package^55^ implemented in R (R Core Team) to calculate a repeated measures correlation between individual model fit and mouse performance to account for the repeated nature of this data (sampling individual mice across days). We used Matlab’s cusum function to get the upper cumulative sum in Fig. 1i, j, using 2 SD as the criterion. In our simple LME models, we used permutation tests comparing actual β coefficient values to a distribution of 1000 order shuffled versions of the same variable, and thus the resolution of our permutation *p* values was *p* < 0.001. We excluded presses over 10 s in duration from all modeling datasets. For event-aligned Ca^2+^ activity comparing Met vs. Fail lever presses, we used permutation tests that required either 4 (for onset and offset-aligned activity) or 3 (for interpolated activity during the press) consecutive samples to pass the threshold for significance. To assess the relationship between Ca^2+^ activity and various aspects of behavior in our complex LME models, we performed F-tests on the individual parameters. For group comparisons (e.g., Sham vs. Lesion) of LME model coefficients, we used *t* tests with Benjamini-Hochberg false discovery rate correction (Q = 5%) on all of the model terms shown in Supplementary Tables 3 and 6. Behavioral data was collected using Med-PC software, analyzed using Excel (Microsoft), Matlab (Mathworks), R (R Core Team), and Prism (Graphpad).

### Reporting summary

Further information on research design is available in the Nature Research Reporting Summary linked to this article.

## Supplementary information

Supplementary information

Editorial Assessment Report

Reporting Summary

## Data Availability

All data supporting the findings of this study are provided within the paper and its Supplementary Information. A source data file is provided with this paper. All additional information will be made available upon request to the authors. Source data are provided with this paper.

## Source data

Source data

## Code availability

The code used to analyze the data from this study is freely available at: https://github.com/gremellab/Hold-Down-Behavior-GCAMP-Opto-analysis.
