# Supplementary material for: Information normally considered task-irrelevant drives decision-making and affects premotor circuit recruitment
Source: Nat Commun. 2022 Apr 19;13:2134. doi: 10.1038/s41467-022-29807-2 (PMC9018678; doi:10.1038/s41467-022-29807-2)
Supplement: Supplementary file 1 — Supplementary information [file 41467_2022_29807_MOESM1_ESM.pdf]

Supplementary Note 1, related to Figure 1. As previous reports have indicated that rats in a similarly unstructured task may sometimes make short, stereotyped lever presses after reward (Platt et al., 1973), we investigated our data for evidence of stereotypies. Of the 13 behavioral mice, we found evidence of 1 mouse that appeared to adopt this stereotyped strategy, making presses after a reward that were 450 ± 434ms (mean ± SD) in duration, while the average for all other animals was 1051 ± 757ms. Permutation tests comparing to order shuffled data found that this same mouse exhibited a smaller SD after a rewarded press in actual versus shuffled data on 7 out of 14 days, while no other mouse in this (or subsequent) experiments did so for more than 2 days. Thus, while it is possible for animals to adopt a stereotyped strategy to perform this task, only a very small minority of animals appear to do so. Aside from the difference in species (rats versus mice), the results of Platt et al. (1973) may have been due to the extensive 2 week pretraining period without a duration requirement, wherein rats would have been incentivized to press as rapidly as possible to earn maximal reward and might develop a habitual or stereotyped response that persisted even after introduction of the duration requirement.

Supplementary Figure 1

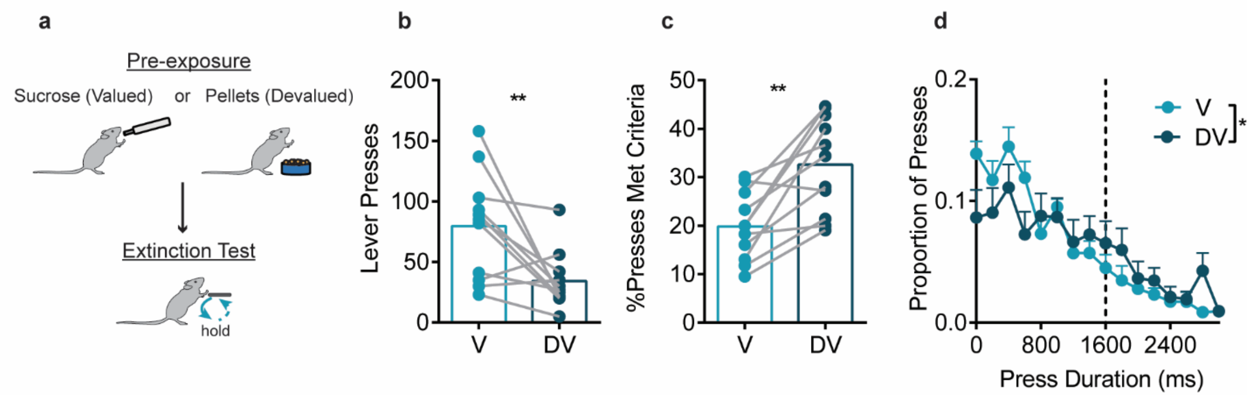

Supplementary Figure 1. *Outcome value has differential control over action selection and execution*, related to Fig. 1. **a** Schematic of the outcome devaluation procedure. In a within-subjects design, mice received 1 hr of pre-exposure to either sucrose (Valued, V) or pellets (Devalued, DV), followed by a 5 minute extinction text across 2 days (order counterbalanced). **b** Total Lever Presses on V and DV days. Paired t-test, $t_{10} = 3.09$, $p = 0.012$. **c** %Presses that met criteria on V and DV days. Paired t-test, $t_{10} = 4.55$, $p = 0.0011$. **d** Histogram of press durations on V and DV days (200 ms bins). 2-way RM ANOVA, main effect of Duration Bin, $F_{15,150} = 12.1$, $p < 0.0001$ and an interaction (Duration Bin x V/DV) $F_{15,150} = 2.19$, $p = 0.009$. All tests were two-tailed with post hoc multiple comparisons correction. Data in d are mean+SEM across mice, bars in b-c are mean. ** $p < 0.01$, * $p < 0.05$. See also Source Data.

Supplementary Figure 2

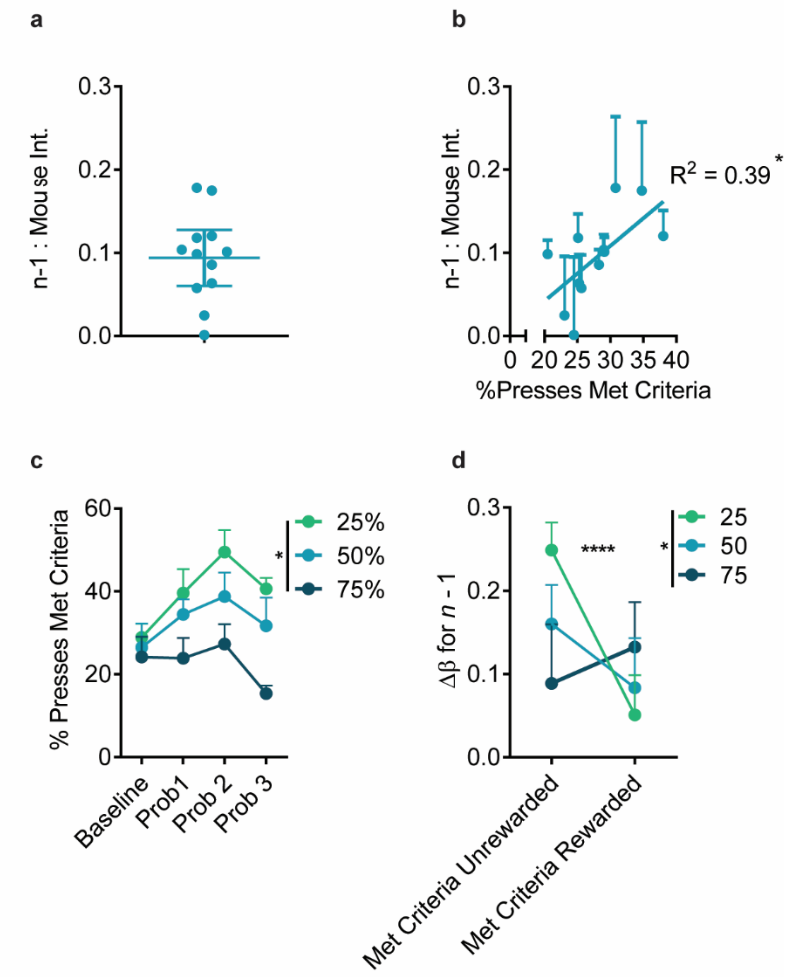

Supplementary Figure 2. I*ndividual variability in use of experience positively correlates with performance, and probabilistic reward induces win-stay behavior*, related to Fig. 2. **a, b** LMEs that predicted the duration of lever press n (as in Fig. 2b) with an additional random interaction term between mouse and n – 1 duration. This allowed us to model how much the use of recent experience (n – 1) varied across individual mice. Here (**a**) we plot the overall β coefficients for individual mice (adding each mouse's interaction term to the overall coefficient). Of these 12 mice, only 4 had significant interactions (mouse X n - 1 Duration). Of these 4 mice (those that lie outside the 95% CI lines in a) half showed an increased relationship, while the other half showed a decreased relationship relative to the overall group mean, suggesting that this relationship is present in the vast majority of mice. **b** There was a significant positive relationship between the individual mouse n – 1 coefficient and performance (indexed via a mouse's average %Presses Met Criteria). Linear Regression: $F_{1,10} = 6.37$, $p = 0.030$, $R^2 = 0.39$.

This suggests that animals who use recent experience to a greater degree may perform more efficiently in the behavior (see also, Fig. 2f). **c, d** Following initial training on 100% reward, mice (n = 15 mice total) were shifted to either 25%, 50%, or 75% (n = 5 mice per probability condition) reward and trained for 3 days. (**c**) %Presses Met Criteria across training. 2-way ANOVA (Probability x Day), no interaction, main effects of Day $F_{3,36} = 6.34$, $p = 0.0015$, and probability group $F_{2,12} = 5.28$, $p = 0.0226$. (**d**) β coefficients for the interaction between presses that met criteria and n – 1 duration. Interaction between n – 1 duration and n – 1 outcome (Met Yes Reward vs. Met No Reward): $F_{4,17403} = 30.2$, $p < 0.0001$, and 3-way interaction between Duration, Outcome, and Group: $F_{4,17403} = 2.59$, $p = 0.035$. Int = Interaction. In c, d n = 15 mice, split into n = 5 mice per probability group. Overall, the probability data suggest that probabilistic reward may induce win-stay behavior, and that mice may use an internal representation of reward, given that this win-stay behavior remains even for Met Criteria Unrewarded lever presses. All tests were two-tailed with post hoc multiple comparisons correction. Data are mean+SEM. Baseline = Final pretraining day with 100% reward. Prob 1, 2, 3 = Training Days 1, 2, or 3 of probability training. **** $p < 0.0001$, * $p < 0.05$. See also Source Data.

Supplementary Figure 3

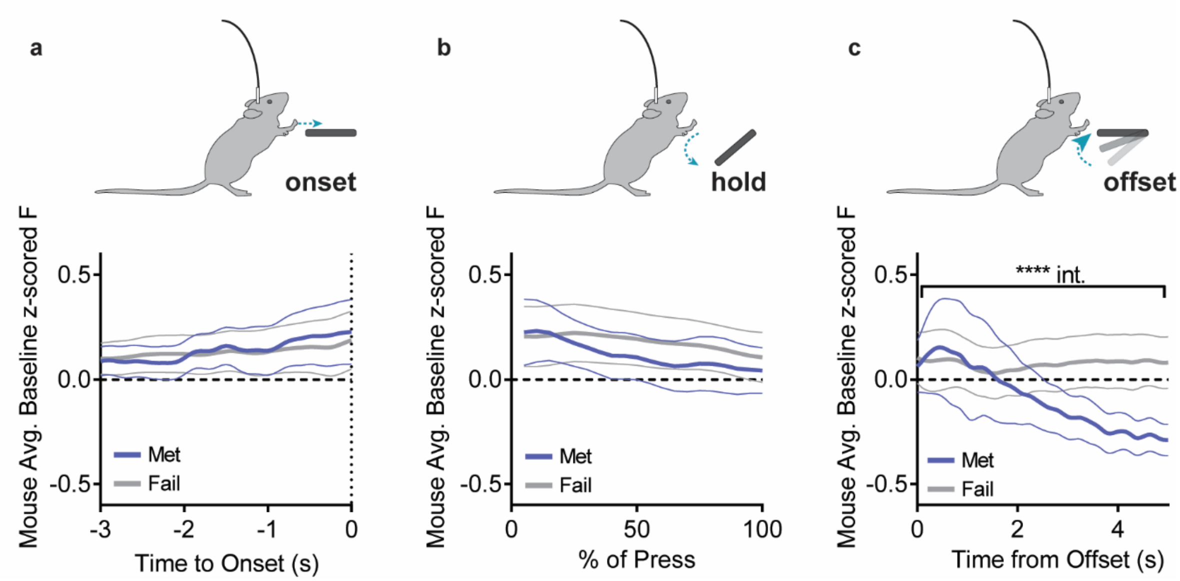

Supplementary Figure 3. *Mouse average M2 Ca2+ activity data shows similar patterns,* related to Fig. 4. **a-c** Instead of collapsing across all mice/days (Fig. 4), here we computed the average Ca2+ activity (z-scored relative to a baseline period) for individual mice across all days of 1600ms training. These mouse average traces were aligned to before press onset (**a**), the duration of the press itself using interpolation (**b**), and after the offset of the lever press (**c**). These mouse average traces revealed largely similar patterns – i.e., ramping activity before press onset, and a difference in Met/Fail activity just after press offset (2-way RM ANOVA Time x Met/Fail, Main effect of Time $F_{100,700} = 1.93$, $p < 0.0001$ and an interaction $F_{100,700} = 2.27$, $p < 0.0001$). All tests were two-tailed with post hoc multiple comparisons correction. Met = Presses that met criterion, Fail = Presses that did not meet criterion. Int. = Significant Interaction. **** = p < 0.0001. See also Source Data.

Supplementary Figure 4

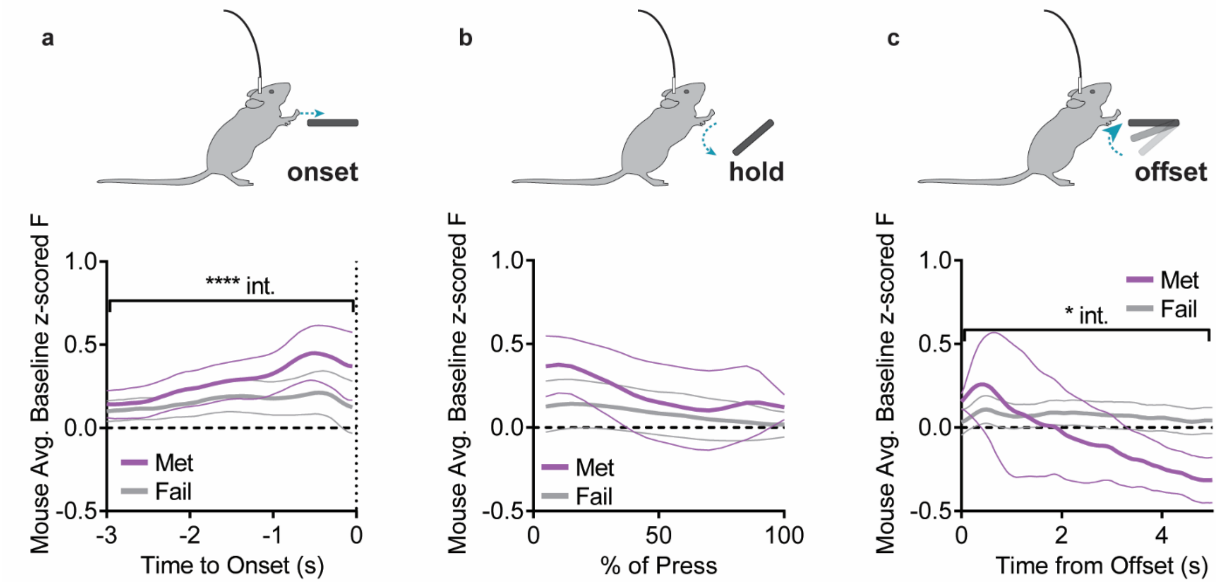

Supplementary Figure 4. *Mouse average M2-DMS Ca2+ activity data shows similar patterns,* related to Fig. 5. **a-c** Instead of collapsing across all mice/days (Fig. 4), here we computed the average Ca2+ activity (z-scored relative to a baseline period) for individual mice across all days of 1600ms training. These mouse average traces were aligned to before press onset (**a**), the duration of the press itself using interpolation (**b**), and after the offset of the lever press (**c**). These mouse average traces revealed largely similar patterns – i.e., ramping activity before press onset which significantly differed between Met/Fail (2-way RM ANOVA Time x Met/Fail, Main effect of Time $F_{59,354} = 1.65$, $p = 0.0032$ and an interaction $F_{59,354} = 3.51$, $p < 0.0001$), and a difference in Met/Fail activity just after press offset (2-way RM ANOVA Time x Met/Fail, Main effect of Time $F_{100,600} = 1.91$, $p < 0.0001$ and an interaction $F_{100,600} = 1.36$, $p = 0.016$). All tests were two-tailed with post hoc multiple comparisons correction. Met = Presses that met criterion, Fail = Presses that did not meet criterion. Int. = Significant Interaction. **** = p < 0.0001, * = p < 0.05. See also source data.

Supplementary Table 1. *Simple LME Model Statistics*, related to Fig. 2.

Term	Coef	SE	Upper	Lower	F-Stat	F-Pval	Perm P
Intercept	223	49.3	320	127	20.2	6.04E-6	N/A
Dur_{n-1}	0.0909	0.00503	0.101	0.081	330	1.16E-72	<0.001
Dur_{n-2}	0.0731	0.00506	0.083	0.0631	207	3.64E-47	<0.001
Dur_{n-3}	0.0543	0.00508	0.0642	0.0443	113	1.48E-26	<0.001
Dur_{n-4}	0.0376	0.0051	0.0476	0.0276	53.9	1.88E-13	<0.001
Dur_{n-5}	0.0228	0.00512	0.0328	0.0127	20.5	8.61E-6	0.007
Dur_{n-6}	0.029	0.00513	0.039	0.0189	31.1	1.63E-8	<0.001
Dur_{n-7}	0.0102	0.00514	0.0203	1.78E-4	3.62	0.0461	0.266
Dur_{n-8}	0.00979	0.00515	0.0199	-0.0003	3.89	0.0572	0.307
Dur_{n-9}	0.0204	0.00514	0.0305	0.0103	15.2	7.35E-5	0.019
Dur_{n-10}	0.00942	0.00513	0.0195	-6.4E-4	3	0.0664	0.328
%Met	12.6	0.533	13.7	11.6	564	3.8E-123	<0.001
Time	3.44E-5	2.84E-6	3.99E-5	2.88E-5	147	1.17E-33	<0.001

Parameters, their coefficients, and statistical tests for the simple LME model predicting *n* duration given n-back durations. Degrees of Freedom for all F-tests = 1, 39711. All tests were two-tailed. Dur_{n-x} = Duration of lever press *n* - x in ms. %Met = %Press Met Criteria. Time = Lever press session timestamp in ms. Coef = β Coefficient. SE = Standard Error. Upper and Lower = 95% confidence intervals. F-stat = F-statistic. F-Pval = p-value from the F-test. Perm P = p-value from permutation test comparing to 1000 order shuffled β coefficients.

Supplementary Table 2. *Complex LME Model Statistics*, Related to Fig. 2.

Term	Coef	SE	Upper	Lower	F-Stat	Pval
Intercept	143	44	229	56.3	10.5	0.0012
Dur_{n-1}	*-0.0405*	*0.0168*	*-0.00763*	*-0.0735*	*5.83*	*0.0158*
Dur_{n-2}	0.0834	0.00609	0.0954	0.0715	188	1.29E-42
Dur_{n-3}	0.0509	0.00501	0.0608	0.0411	104	2.72E-24
Dur_{n-4}	0.0351	0.00502	0.045	0.0253	48.9	2.7E-12
Dur_{n-5}	0.0188	0.00503	0.0286	0.0089	13.9	1.91E-4
Dur_{n-6}	0.0279	0.00502	0.0377	0.0181	30.9	2.75E-8
MA	0.354	0.0412	0.435	0.273	73.8	8.85E-18
HE_{n-1}	-79.5	23.1	-34.3	-125	11.9	5.7E-4
Dur_{n-1} : HE_{n-1}	0.0656	0.0118	0.0888	0.0425	30.9	2.72E-8
MA : HE_{n-1}	0.219	0.0276	0.273	0.165	62.9	2.23E-15
Rew_{n-1}	*-60.6*	*29.7*	*-2.29*	*-119*	*4.15*	*0.0416*
Dur_{n-1} : Rew_{n-1}	*0.00994*	*0.0151*	*0.0395*	*-0.0196*	*0.436*	*0.509*
MA : Rew_{n-1}	*-0.0414*	*0.0314*	*0.0201*	*-0.103*	*1.74*	*0.187*
IPI_{n-1}	*7.11E-4*	*3.4E-4*	*0.00138*	*4.34E-5*	*4.36*	*0.0368*
Dur_{n-1} : IPI_{n-1}	-7.9E-7	1.25E-7	-5.4E-7	-1E-6	39.7	2.92E-10
MA : IPI_{n-1}	*-4.1E-8*	*3.34E-7*	*6.13E-7*	*-7E-7*	*0.0153*	*0.902*
IPI_{n-2}	*2.15E-6*	*1.36E-4*	*2.69E-4*	*-0.00027*	*2.48E-4*	*0.987*
Dur_{n-2} : IPI_{n-2}	-3.3E-7	7.72E-8	-1.8E-7	-4.8E-7	18.3	1.89E-5
Time	1.51E-5	7.58E-6	3.0E-5	2.85E-7	3.99	0.0458
Dur_{n-1} : Time	2.16E-8	3.94E-9	2.93E-8	1.39E-8	30	4.37E-8
MA : Time	*-8.3E-9*	*8.85E-9*	*9.01E-9*	*-2.6E-8*	*0.887*	*0.346*
%Met	10.9	1.13	13.1	8.64	92.5	7E-22
Dur_{n-1} : %Met	0.00271	4.3E-4	0.00355	0.00187	39.7	3E-10
MA : %Met	-0.00491	0.00113	-0.0027	-0.00713	18.9	1.42E-5

Parameters, their coefficients, and statistical tests for the Complex LME model. Degrees of Freedom for all F-tests = 1, 40203. All tests were two-tailed. Some parameters did not improve the model by BIC, but were included in the model because: 1) null effects are of interest (e.g., Rew_{n-1}), 2) non-significant main effects were kept if their interactions were significant (e.g., IPI_{n-2}) and 3) we kept the same interaction terms for both Dur_{n-1} and the MA to determine if they were differentially controlled by experiential variables (e.g., MA : Time). These BIC non-significant terms are italicized. Dur_{n-x} = Duration of press n - x. MA = Moving Average. HE_{n-1} = Headentry between presses n and n - 1. Rew_{n-1} = n - 1 reward. Data presented here for these two binary variables indicates the effect if they occur (e.g., the interaction between Dur_{n-1} : HE_{n-1} shows how the contribution of Duration changes given that a HE occurred in between press n and press n - 1). IPI_{n-x} = IPI between press n and press n - x. Time = Time in session that a lever press occurred. %Met = % of presses that met the duration criterion for a given session. Coef = β Coefficient. SE = Standard Error. Upper and Lower = 95% confidence intervals. F-stat = F-statistic. Pval = p-value from the F-test.

Supplementary Table 3. *M2 Sham/Lesion Complex LME Model Statistics*, Related to Fig. 3.

	Sham (n = 24630, df = 24605)				Lesion (n = 33348, df = 33323)			
Term	Coef	SE	F	Pval	Coef	SE	F	Pval
Intercept	266	58.5	20.7	5.5E-6	186	46.3	16.1	6.08E-5
**Dur$_{n-1}$**	**-0.0738**	**0.0221**	**11.1**	**8.59E-4**	**0.0708**	**0.0185**	**14.6**	**1.32E-4**
Dur$_{n-2}$	0.0523	0.00767	46.5	9.43E-12	0.0516	0.00621	69.2	9.34E-17
Dur$_{n-3}$	0.0428	0.00638	45.1	1.88E-11	0.0528	0.00552	91.4	1.24E-21
**Dur$_{n-4}$**	**0.0408**	**0.00638**	**40.9**	**1.61E-10**	**0.0158**	**0.00553**	**8.19**	**0.00422**
Dur$_{n-5}$	0.0168	0.00639	6.94	0.00843	0.0302	0.00553	29.9	4.61E-8
Dur$_{n-6}$	0.0195	0.0064	9.26	0.00234	0.0279	0.00551	25.6	4.14E-7
MA	0.244	0.0566	18.6	1.66E-5	0.216	0.0445	23.7	1.13E-6
HE$_{n-1}$	-43.4	25.3	2.93	0.0868	-22.3	22.7	0.969	0.325
**Dur$_{n-1}$: HE$_{n-1}$**	**0.0635**	**0.0153**	**17.2**	**3.39E-5**	**-0.0149**	**0.0131**	**1.3**	**0.254**
MA : HE$_{n-1}$	0.149	0.032	21.8	3.01E-6	0.165	0.0291	32.2	1.42E-8
Rew$_{n-1}$	42.3	33	1.64	0.2	-41.6	31	1.8	0.179
**Dur$_{n-1}$: Rew$_{n-1}$**	**-0.065**	**0.0192**	**11.4**	**7.21E-4**	**0.0014**	**0.0168**	**6.91E-3**	**0.934**
MA : Rew$_{n-1}$	-0.101	0.0375	7.25	0.00708	-0.0354	0.0347	1.04	0.307
IPI$_{n-1}$	-1.2E-4	4.41E-4	0.0694	0.792	-1.29E-3	4.11E-4	9.83	0.00172
**Dur$_{n-1}$: IPI$_{n-1}$**	**-6.7E-07**	**1.6E-7**	**17.7**	**2.58E-5**	**-1.2E-7**	**7.99E-8**	**2.24**	**0.134**
MA : IPI$_{n-1}$	9E-7	4.22E-7	4.55	0.0329	1.63E-6	3.98E-7	16.7	4.29E-5
IPI$_{n-2}$	-0.0005	1.55E-4	10.6	0.00116	-4.4E-4	1.31E-4	11.4	7.31E-4
Dur$_{n-2}$: IPI$_{n-2}$	-2.3E-7	8.2E-8	8.05	0.00454	-3.3E-8	5.11E-8	0.421	0.516
Time	2.33E-5	8.27E-6	7.93	0.00486	2.03E-5	7.39E-6	7.53	0.00607
**Dur$_{n-1}$: Time**	**3.18E-8**	**4.95E-9**	**41.2**	**1.39E-10**	**1.26E-8**	**4.1E-9**	**9.44**	**0.00213**
MA : Time	-1.7E-8	9.63E-9	3.03	0.0815	-1.1E-8	8.47E-9	1.8	0.18
%Met	8.32	1.52	30	3.97E-8	8.03	1.18	46.6	8.82E-12
**Dur$_{n-1}$: %Met**	**0.00382**	**5.96E-4**	**41**	**1.54E-10**	**8.22E-4**	**4.44E-4**	**3.42**	**0.0644**
MA : %Met	-8.6E-4	0.0016	0.293	0.589	0.00179	0.00119	2.28	0.131

Parameters, their coefficients, and statistical tests for the Complex LME models from M2 Sham and M2 Lesion groups. Bolded terms denote significant Sham/Lesion group differences,

assessed using two-tailed unpaired t-tests with Benjamini-Hockberg false discovery correction as follows. DF for all the following tests are 57976. Dur_{n-1}; t = 5.03, p < 0.0001. Dur_{n-4}; t = 2.96, p = 0.003. Dur_{n-1} : HE_{n-1}; t = 3.89, p < 0.0001. Dur_{n-1} : Rew_{n-1}; t = 2.59 , p = 0.00951. Dur_{n-1} : IPI_{n-1}; t = 3.34, p = 0.000845. Dur_{n-1} : Time; t = 3.00, p = 0.00267. Dur_{n-1} : %Met; t = 4.12 , p < 0.0001. Dur_{n-x} = Duration of press n - x. MA = Moving Average. HE_{n-1} = Headentry between presses n and n - 1. Rew_{n-1} = n - 1 reward. IPI_{n-x} = IPI between press n and press n - x. Time = Time in session that a lever press occurred. %Met = % of presses that met the duration criterion for a given session. Coef = β Coefficient. SE = Standard Error. Upper and Lower = 95% confidence intervals. F = F-statistic. Pval = p-value from the F-test.

Supplementary Table 4, *M2 GCaMP Complex LME Model Statistics*, related to Fig. 4.

	Before Press, df = 11359			During Press, df = 6759			After Press, df = 11359		
Term	Coef	F	p	Coef	F	p	Coef	F	p
Int.	2.26	1.47	0.225	0.82	0.131	0.718	-1.76	0.704	0.402
Dur$_{n}$	**-4.0E-4**	**4.86**	**0.0275**	**-1.95E-3**	**75.6**	**4.26E-18**	-2.3E-4	0.249	0.618
Dur$_{n-1}$	**-1.6E-3**	**5.34**	**0.0209**	9.04E-4	0.933	0.334	-3.2E-4	0.198	0.656
Dur$_{n-2}$	**6.35E-4**	**9.16**	**0.00248**	**6.21E-4**	**5.6**	**0.018**	**5.24E-4**	**5.75**	**0.0165**
Dur$_{n-3}$	1.0E-4	0.302	0.583	-2.6E-4	1.35	0.245	2.3E-4	1.46	0.226
Dur$_{n-4}$	2.61E-4	2.02	0.155	**4.62E-4**	**4.15**	**0.0416**	2.77E-4	2.1	0.147
Dur$_{n-5}$	6.81E-5	0.137	0.711	-4.7E-5	0.0437	0.834	7.87E-5	0.169	0.681
Dur$_{n-6}$	2.1E-4	1.31	0.253	2.5E-4	1.22	0.269	3.21E-4	2.8	0.0942
MA	-1.49E-3	0.699	0.403	2.2E-4	0.0086	0.926	0.00138	0.547	0.46
HE$_{n-1}$	**8.02**	**25.2**	**5.3E-7**	**8.29**	**14.8**	**1.19E-4**	2.45	2.15	0.142
Dur$_{n-1}$: HE$_{n-1}$	**0.00241**	**24.6**	**7.13E-7**	**0.00217**	**13.4**	**2.48E-4**	**0.00269**	**28.1**	**1.15E-7**
MA : HE$_{n-1}$	**-6.27E-3**	**14.6**	**1.35E-4**	**-5.91E-3**	**8.15**	**0.00433**	-2.88E-3	2.84	0.0919
Rew$_{n-1}$	-1.65	0.686	0.407	-0.0339	1.79E-4	0.989	1.03	0.247	0.619
Dur$_{n-1}$: Rew$_{n-1}$	**0.00241**	**14.6**	**1.34E-4**	0.00128	2.49	0.115	1.22E-4	0.0346	0.852
MA : Rew$_{n-1}$	1.13E-4	0.00482	0.945	01.98E-4	0.00987	0.921	0.00197	1.37	0.242
IPI$_{n-1}$	**7.05E-5**	**15.8**	**7.14E-5**	**5.86E-5**	**6.59**	**0.0103**	**6.39E-5**	**12**	**5.4E-4**
Dur$_{n-1}$: IPI$_{n-1}$	**-1.3E-8**	**6.07**	**0.0138**	-1.2E-8	3.72	0.0537	**-1.5E-8**	**7.21**	**0.00727**
MA : IPI$_{n-1}$	-2.8E-8	3.34	0.0678	-2.2E-8	1.26	0.263	-2.3E-8	2.04	0.154
IPI$_{n-2}$	2.19E-6	0.0729	0.787	7.99E-6	0.702	0.402	5.4E-6	0.41	0.522
Dur$_{n-2}$: IPI$_{n-2}$	-4.9E-9	1.04	0.308	**-1.2E-8**	**4.64**	**0.0313**	**-1E-8**	**4.24**	**0.0395**
Time	**-1.2E-6**	**9.62**	**0.00193**	-7.1E-7	1.69	0.194	**-1.2E-6**	**9.96**	**0.0016**
Dur$_{n-1}$: Time	-7E-11	0.234	0.628	-1.7E-10	0.843	0.359	-1E-10	0.468	0.494
MA : Time	**1.01E-9**	**5.48**	**0.0192**	7.68E-10	1.7	0.193	**1.3E-9**	**8.37**	**0.00382**
%Met	-0.025	0.174	0.676	-0.0268	0.107	0.743	0.0409	0.412	0.521

Dur_{n-1} : %Met	-3.2E-5	1.77	0.184	-5.6E-5	3.48	0.0621	-1.4E-5	0.293	0.588
MA : %Met	5.27E-5	0.913	0.339	6.09E-5	0.727	0.394	-4.2E-5	0.491	0.483
Act_{n-1}	**0.453**	**2370**	**0**	**0.393**	**1070**	**1.3E-217**	**0.431**	**2130**	**0**
Act_{n-2}	-3.77E-3	0.138	0.711	0.0202	2.48	0.115	0.0106	1.1	0.295
Act_{n-3}	**0.0251**	**6.07**	**0.0138**	-0.0186	2.09	0.148	-3.66E-3	0.131	0.717
Act_{n-4}	-5.33E-3	0.274	0.601	0.0208	2.59	0.108	**0.0481**	**22.6**	**2.0E-6**
Act_{n-5}	0.0134	1.74	0.187	0.00597	0.216	0.642	**-0.0215**	**4.52**	**0.0336**
Act_{n-6}	0.0129	1.93	0.165	0.017	2.01	0.157	**0.0259**	**7.8**	**0.00524**
Rew_n	NA	NA	NA	NA	NA	NA	**5.79**	**13.4**	**2.52E-4**
Dur_n : Rew_n	NA	NA	NA	NA	NA	NA	8.35E-4	2.04	0.153
MA : Rew_n	NA	NA	NA	NA	NA	NA	**-7.64E-3**	**29.8**	**4.99E-8**

Parameters, their coefficients, and statistical tests relating M2 calcium activity to behavior. All tests were two-tailed. We predicted activity at three different time points -1s to 0s Before Press, During the Press, and 0s to +1s After Press offset. We included prior activity (Act_{n-x}) as a covariate to control for autocorrelation in calcium activity data. Bolded terms are significant by F-test on the model. In the After Press model, we also incorporated an n - 0 reward term (i.e., was the just completed press rewarded) given that we see an apparent reward response at this time point (Fig. 4d). P-values of 0 are reported for some prior activity terms due to Matlab's numerical resolution. Dur_{n-x} = Duration of press n - x. MA = Moving Average. HE_{n-1} = Headentry between presses n and n - 1. Rew_{n-1} = n - 1 reward. IPI_{n-x} = IPI between press n and press n - x. Time = Time in session that a lever press occurred. %Met = % of presses that met the duration criterion for a given session. Coef = β Coefficient. SE = Standard Error. Upper and Lower = 95% confidence intervals. F = F-statistic. p = p-value from the F-test.

Supplementary Table 5. *M2-DMS GCaMP Complex LME Model Statistics*, related to Fig. 5.

	Before Press, df = 12038			During Press, df = 7524			After Press, df = 12025		
Term	Coef	F	p	Coef	F	p	Coef	F	p
Int.	-0.501	0.0655	0.798	-0.461	0.0273	0.869	-2.06	0.815	0.367
Dur_{n-0}	**6.79E-4**	**11.2**	**8.22E-4**	-3.1E-4	1.42	0.234	**0.00247**	**16.8**	**4.15E-5**
Dur_{n-1}	-1.48E-3	3.22	0.0727	8.22E-5	0.00508	0.943	-1.49E-3	2.58	0.108
Dur_{n-2}	-2.4E-5	0.0102	0.92	1.69E-4	0.283	0.594	4.82E-5	0.0318	0.858
Dur_{n-3}	6.01E-6	8.58E-4	0.977	-3.1E-5	0.0134	0.908	1.36E-4	0.349	0.554
Dur_{n-4}	-1.4E-4	0.435	0.51	-6.1E-5	0.0526	0.819	3.41E-4	2.19	0.139
Dur_{n-5}	-1.1E-4	0.261	0.61	-1.6E-4	0.348	0.555	-9.6E-6	0.00172	0.967
Dur_{n-6}	-4.3E-5	0.0426	0.837	2.37E-4	0.789	0.375	-9.8E-5	0.179	0.672
MA	-2.12E-3	1.08	0.298	-5.08E-3	3.06	0.0804	-3.5E-3	2.33	0.127
HE_{n-1}	**4.49**	**4.29**	**0.0382**	**6.41**	**4.09**	**0.0432**	0.751	0.0957	0.757
Dur_{n-1} : HE_{n-1}	**0.00126**	**4.91**	**0.0268**	**0.00213**	**8.67**	**0.00325**	**0.00136**	**4.53**	**0.0334**
MA : HE_{n-1}	5.42E-5	6.85E-4	0.979	-2.34E-3	0.627	0.429	0.00404	3.02	0.0821
Rew_{n-1}	**6.91**	**7.19**	**0.00732**	5.72	2.69	0.101	**9.31**	**10.4**	**0.00127**
Dur_{n-1} : Rew_{n-1}	-9.5E-4	1.67	0.196	-6.3E-4	0.425	0.514	2.71E-4	0.108	0.742
MA : Rew_{n-1}	-1.55E-3	0.514	0.473	-5.6E-4	0.0371	0.847	-2.79E-3	1.34	0.248
IPI_{n-1}	**-7.2E-5**	**13.6**	**2.3E-4**	**-6.7E-5**	**4.76**	**0.0292**	**-8.8E-5**	**16.2**	**5.66E-5**
Dur_{n-1} : IPI_{n-1}	-1.7E-9	0.0838	0.772	-3.3E-9	0.178	0.673	7.63E-10	0.0136	0.907
MA : IPI_{n-1}	**1.17E-7**	**40.3**	**2.25E-10**	**1.21E-7**	**17.4**	**3.12E-5**	**1.01E-7**	**23.9**	**1.0E-6**
IPI_{n-2}	-1.5E-5	3.21	0.0732	6.6E-7	0.00335	0.954	-4.5E-6	0.238	0.626
Dur_{n-2} : IPI_{n-2}	2.62E-9	0.32	0.572	4.26E-9	0.462	0.497	8.68E-11	2.8E-4	0.987
Time	**1.31E-6**	**8.42**	**0.00371**	-2E-8	8.36E-4	0.977	**1.37E-6**	**7.28**	**0.00698**
Dur_{n-1} : Time	-1.3E-10	0.695	0.405	-9.8E-11	0.238	0.626	-3.3E-10	3.74	0.0531
MA : Time	**-1.5E-9**	**8.25**	**0.00409**	1.03E-11	1.96E-4	0.989	**-1.3E-9**	**5.45**	**0.0196**
%Met	0.0512	0.282	0.595	0.194	2.22	0.136	-0.0109	0.00966	0.922

Dur_{n-1} : %Met	5.03E-5	3.06	0.0803	-3.3E-5	0.784	0.376	2.22E-6	0.00471	0.945
MA : %Met	7.62E-5	0.884	0.347	2.64E-5	0.056	0.813	**1.93E-4**	**4.28**	**0.0387**
Act_{n-1}	**0.387**	**1850**	**0**	**0.342**	**893**	**1.4E-185**	**0.321**	**1250**	**1.8E-260**
Act_{n-2}	**0.0254**	**7.02**	**0.00806**	**0.0322**	**7.25**	**0.00712**	**0.0298**	**9.89**	**0.00167**
Act_{n-3}	**0.0316**	**10.9**	**9.72E-4**	**0.0431**	**13**	**3.2E-4**	0.0162	2.94	0.0866
Act_{n-4}	**0.0438**	**21**	**4.56E-6**	**0.0384**	**10.3**	**0.00131**	**0.0213**	**5.08**	**0.0242**
Act_{n-5}	0.0111	1.34	0.247	0.0202	2.85	0.0913	-9.24E-3	0.96	0.327
Act_{n-6}	**0.0465**	**27.5**	**1.64E-7**	**0.0653**	**33.5**	**7.59E-9**	**0.0418**	**21.7**	**3.24E-6**
Rew_n	NA	NA	NA	NA	NA	NA	**10.1**	**19**	**1.31E-5**
Dur_n : Rew_n	NA	NA	NA	NA	NA	NA	**-3.22E-3**	**18.1**	**2.06E-5**
MA : Rew_n	NA	NA	NA	NA	NA	NA	**-7.16E-3**	**12.4**	**4.29E-4**

Parameters, their coefficients, and statistical tests relating M2 calcium activity to behavior. All tests were two-tailed. We predicted activity at three different time points -1s to 0s Before Press, During the Press, and 0s to +1s After Press offset. We included prior activity (Act_{n-x}) as a covariate to control for autocorrelation in calcium activity data. Bolded terms are significant by F-test on the model. In the After Press model, we also incorporated an n - 0 reward (i.e., was the just completed press rewarded) term given that we see an apparent reward response at this time point (Fig. 5d). P-values of 0 are reported for prior activity due to Matlab's numerical resolution. Dur_{n-x} = Duration of press n - x. MA = Moving Average. HE_{n-1} = Headentry between presses n and n - 1. Rew_{n-1} = n - 1 reward. IPI_{n-x} = IPI between press n and press n - x. Time = Time in session that a lever press occurred. %Met = % of presses that met the duration criterion for a given session. Coef = β Coefficient. SE = Standard Error. Upper and Lower = 95% confidence intervals. F = F-statistic. p = p-value from the F-test.

Supplementary Table 6. *M2-DMS Sham/Lesion Complex LME Model Statistics*, related to Fig. 6.

	Sham (n = 23758 df = 23733)				Lesion (n = 23596, df = 23571)			
Term	Coef	SE	F	Pval	Coef	SE	F	Pval
Int.	291	55	28	1.2E-7	285	58	24.1	9.17E-7
Dur_{n-1}	-0.0284	0.0212	1.8	0.18	-0.0313	0.0217	2.08	0.149
Dur_{n-2}	0.0607	0.00763	63.3	1.83E-15	0.0691	0.00772	80.2	3.68E-19
Dur_{n-3}	0.0229	0.00655	12.3	4.64E-4	0.0434	0.0066	43.2	5.03E-11
Dur_{n-4}	0.0199	0.00656	9.25	0.00235	0.0308	0.00661	21.7	3.13E-6
Dur_{n-5}	0.0301	0.00657	21	4.69E-7	0.0415	0.00662	39.3	3.79E-10
Dur_{n-6}	0.0282	0.00658	18.3	1.86E-6	0.017	0.00664	6.54	0.0105
MA	0.217	0.0561	15	1.08E-4	0.2	0.0585	11.7	6.23E-4
HE_{n-1}	-79.1	28.6	7.66	0.00565	-38.2	31.2	1.5	0.221
**Dur_{n-1} : HE_{n-1}**	**0.0295**	**0.0157**	**3.52**	**0.0607**	**-0.0412**	**0.0165**	**6.22**	**0.0126**
MA : HE_{n-1}	0.0959	0.0366	6.86	0.00882	0.157	0.0399	15.6	7.94E-5
Rew_{n-1}	-73.2	37.7	3.77	0.0523	-77.3	37.5	4.25	0.0392
Dur_{n-1} : Rew_{n-1}	-0.0349	0.0194	3.22	0.0725	-0.00192	0.0193	0.00983	0.921
MA : Rew_{n-1}	0.0708	0.0432	2.68	0.101	0.112	0.0464	5.8	0.0161
IPI_{n-1}	0.00187	4.68E-4	16	6.32E-5	7.19E-4	4.03E-4	3.18	0.0748
Dur_{n-1} : IPI_{n-1}	-6.3E-7	1.49E-7	17.9	2.36E-5	-5.2E-7	1.62E-7	10.4	0.00129
MA : IPI_{n-1}	-8.3E-7	4.75E-7	3.04	0.0813	-2.4E-8	4.04E-7	0.00362	0.952
IPI_{n-2}	-4.8E-4	1.63E-4	8.79	0.00304	-4.3E-4	1.59E-4	7.25	0.00709
Dur_{n-2} : IPI_{n-2}	-5.8E-8	9.35E-8	0.391	0.532	-1.2E-7	9.64E-8	1.46	0.227
Time	-5.2E-6	1.02E-5	0.261	0.61	-9.2E-7	9.78E-6	0.00877	0.925
Dur_{n-1} : Time	3.59E-8	4.93E-9	52.9	3.59E-13	3.39E-8	5.04E-9	45.2	1.81E-11
MA : Time	8.68E-9	1.17E-8	0.553	0.457	1.43E-9	1.21E-8	0.014	0.906
%Met	7.95	1.37	33.8	6.29E-9	7.74	1.55	25	5.7E-7
Dur_{n-1} : %Met	0.00276	5.12E-4	29	7.22E-8	0.00105	6.01E-4	3.06	0.0804
MA : %Met	-0.0013	0.00146	0.792	0.374	-1.3E-4	0.00172	0.00543	0.941

Parameters, their coefficients, and statistical tests for the Complex LME models from M2-DMS Sham and Lesion groups. Bolded terms denote significant Sham/Lesion group differences, assessed using two-tailed unpaired t-tests with Benjamini-Hockberg false discovery correction. Only the Dur_{n-1} : HE_{n-1} interaction significantly differed between Sham and Lesion; t_{47352} = 3.10, p

= 0.00193. Dur_{n-x} = Duration of press n - x. MA = Moving Average. HE_{n-1} = Headentry between presses n and n - 1. Rew_{n-1} = n - 1 reward. IPI_{n-x} = IPI between press n and press n - x. Time = Time in session that a lever press occurred. %Met = % of presses that met the duration criterion for a given session. Coef = β Coefficient. SE = Standard Error. Upper and Lower = 95% confidence intervals. F = F-statistic. Pval = p-value from the F-test.
