# Supplementary material for: Information normally considered task-irrelevant drives decision-making and affects premotor circuit recruitment
Source: Nat Commun. 2022 Apr 19;13:2134. doi: 10.1038/s41467-022-29807-2 (PMC9018678; doi:10.1038/s41467-022-29807-2)
Supplement: Supplementary file 2 — Editorial Assessment Report [file 41467_2022_29807_MOESM2_ESM.pdf]

Contents of this report

- **Manuscript details**: overview of your manuscript and the editorial team.
- **Review synthesis**: summary of the reviewer reports provided by the editors.
- **Editorial recommendation**: personalized evaluation and recommendation from all 3 journals.
- **Annotated reviewer comments**: the referee reports with comments from the editors.
- **Open research evaluation**: advice for adhering to best reproducibility practices.

About the editorial process

Because you selected the **Nature Portfolio Guided Open Access option**, your manuscript was assessed for suitability in three of our titles publishing high-quality work across your field of research. More information about Guided Open Access can be found **here**.

Collaborative editorial assessment

Your editorial team discussed the manuscript to determine its suitability for the Nature Portfolio Guided OA pilot. Our assessment of your manuscript takes into account several factors, including whether the work meets the **technical standard** of the Nature Portfolio and whether the findings are of **immediate significance** to the readership of at least one of the participating journals in the Guided OA pilot.

Peer review

Experts were asked to evaluate the following aspects of your manuscript:

- **Novelty** in comparison to prior publications;
- **Likely audience** of researchers in terms of broad fields of study and size;
- **Potential impact** of the study on the immediate or wider research field;
- **Evidence** for the claims and whether additional experiments or analyses could feasibly strengthen the evidence;
- **Methodological detail** and whether the manuscript is reproducible as written;
- Appropriateness of the literature review.

Editorial evaluation of reviews

Your editorial team discussed the potential suitability of your manuscript for each of the participating journals. They then discussed the revisions necessary in order for the work to be published, keeping each journal's specific editorial criteria in mind.

Journals in the Nature portfolio will support authors wishing to transfer their reviews and (where reviewers agree) the reviewers' identities to journals outside of Springer Nature.

If you have any questions about review portability, please contact our editorial office at guidedoa@nature.com.

Manuscript details

Tracking number	Submission date	Decision date
GUIDEDOA-21-00186	8 July 2021	21 September 2021

**Title**	Mice are not automatons; subjective experience in premotor circuits guides behavior	**Corresponding author**	Christina Gremel<br>**Affiliation:** University of California, San Diego
**Preprint information**	There is a preprint of this manuscript posted at https://www.biorxiv.org/content/10.1101/2021.06.23.449617v1	**Peer review type**	Single-blind

Editorial assessment team

**Primary editor**	**Luis Mejia**<br>**Home Journal:** *Nature Neuroscience*, ORCID: 0000-0001-5439-6803<br>**Email:** luis.mejia@us.nature.com
**Editorial team members**	**David Rowland**, *Nature*, ORCID: 0000-0002-2735-2730<br>**Christian Schnell**, *Nature Communications*, ORCID: 0000-0002-3499-9217
**About your primary editor**	Luis joined Nature Neuroscience in 2018. He received his Ph.D. in Neuroscience from Harvard University/Harvard Medical School, followed by postdoctoral research at Cold Spring Harbor Laboratory in the lab of Bo Li, where he studied orbitofrontal-striatal projection neurons in value and valence based decisions and behaviors in mice, using in vivo optogenetics and calcium imaging. His research interests include systems and circuits neuroscience, reward and aversion learning, and in vivo imaging and neuroscience methods. Luis is based in the New York office.

Editorial assessment and review synthesis

**Editor's summary and assessment**	The authors examine the influence of subjective experiential variables on decision making behavior on a self-paced, self-initiated free foraging lever press task and the role of M2-DMS circuits, by applying behavioral analyses and modeling, causal manipulations and fiber photometry data. The editors found the work to be timely and of interest. The editors also had questions about the level of novelty and advance in the present findings and the data support for the conclusions.<br><br>As part of the Guided Open Access pilot, editors from Nature, Nature Neuroscience, and Nature Communications have discussed the reviewer reports and the manuscript's suitability for the journals. After careful evaluation, our editorial recommendation is to revise the manuscript and submit back through the Guided Open Access submission portal for consideration at Nature Communications.
**Editorial synthesis of reviews**	Your manuscript has been seen by 3 reviewers with expertise in decision making behaviors, reward learning, cortical and basal ganglia circuits, and in vivo imaging/recordings. While the reviewers find the work to be of interest, they have raised substantial concerns about the data support for the conclusions and the conceptual advance provided in the new results.<br><br>Altogether, these critiques preclude continued consideration by Nature and Nature Neuroscience. However, Nature Communications would be prepared to consider a revised manuscript that, at a minimum, addresses the technical and conceptual concerns including the following revisions:<br><br>1) Address the comments from each of the referees regarding the framing of the study as it pertains to 'subjective experience', and its context and placement relative to the previous literature.<br>2) Address reviewers' 1 and 3 comments on the fiber photometry data.<br>3) Provide further analysis of individual mice as suggested by reviewer 2, to strengthen the support for conclusions.<br>4) Address reviewer 2's comments about the methods/analyses reporting; provide analyses applying the LME model to 800 msec criteria sessions, to strengthen support for conclusions.<br>5) Address reviewer 3's points regarding statistical tests with new analyses, to strengthen the support for conclusions.<br>6) Discuss the reviewers' comments on the novelty and conceptual advance provided.

Editorial recommendation

**Nature**	**Revision not invited**	Following editorial assessment of the paper and reviewer reports it was felt that the conceptual advance is not sufficient for further consideration at Nature.
**Nature Neuroscience**	**Revision not invited**	Following editorial assessment of the paper and reviewer reports it was felt that the conceptual advance and level of support for the conclusions is not sufficient for further consideration at Nature Neuroscience.
**Nature Communications**	**Major revisions**	Given the reviewers' concerns, the editors agree it would be necessary to clarify the framing of the study, clarify the novelty and advance, and address all of the technical and methodological concerns.

Next steps

Recommendation Summary

- Option 1: Revise for consideration at Nature Communications

See the previous page for details

Revision

If you would like to follow our recommendation, please upload the revised manuscript, along with your point-by-point response to the reviewers' reports and editorial advice **using the link provided in the decision letter**.

Revision checklist

- Cover letter, stating to which journal you are submitting
- Revised manuscript
- Point-by-point response to reviews
- Updated **Reporting Summary** and **Editorial Policy Checklist**
- Supplementary materials (if applicable)

Submission elsewhere

Within the Nature Portfolio

Springer Nature provides authors with the ability to transfer a manuscript within the Nature Portfolio, without the author having to upload the manuscript data again. To use this service, please **follow the transfer link provided in the decision letter**.

Note that any decision to opt in to *In Review* at the original journal is not sent to the receiving journal on transfer. You can opt in to *In Review* at receiving journals that support this service by choosing to modify your manuscript on transfer.

To a journal outside of Nature Portfolio

We can share the reviews with another journal outside of the Nature Portfolio if requested. You will need to request that the receiving journal office contacts us at guidedOA@nature.com. We have included editorial guidance below in the reviewer reports and open research evaluation to aid in revising the manuscript for publication elsewhere.

Annotated reviewer reports

The editors have included some additional comments on specific points raised by the reviewers below, to clarify requirements for publication in the recommended journal(s). However, please note that all points should be addressed in a revision, even if an editor has not specifically commented on them.

**Reviewer #1**	
**Reviewer #1**	This reviewer has not chosen to waive anonymity. The reviewer's identity can only be shared with representatives of an established journal editorial office.
**Reviewer #1 expertise**<br>Summarised by the editor	Decision making, learning, basal ganglia circuits, in vivo imaging/recordings
**Editor's comments about this review**	The reviewer has provided an overall positive assessment of the paper, but has raised important concerns regarding the conceptual framing and advance, and technical aspects related to the support of the insight and conclusions. Please see also major comments 1, 2, and 5.
**Reviewer #1 comments**	
**Overview**	Remarks to the Author: Overall significance<br>The authors trained mice on a self-paced lever press hold down task. They first used multiple linear regressions to explore which factors influence press duration. Among other correlations, the authors find that 1) head entry into the food magazine was associated with increased similarity between subsequent press durations, 2) the relationship between subsequent presses decreased as the inter-press-interval increases. M2 lesioned mice executed lever press durations that were more similar to their prior action, eliminating some of these other modulators of decisions that occurred without the lesion. Previous lever press durations predicted subsequent M2 and M2-DMS fiber photometry activity. Lesions or acute inhibition of M2-DMS disrupted influence of recent experience in guiding lever press duration but otherwise leaves performance unaffected. While I do have some major concerns, as described below, I think that central finding- that M2 activity correlates with "off-task" variables, and lesioning M2 eliminates the correlation between those variables and press- really is a neat result.<br>Remarks to the Author: Impact<br>I think the paper could be of interest, at least at people interested in the brain areas

	under investigation (M2 & DMS), if the framing could be clarified. I do think they have a clear result that as the authors say may relate to a role of these structures in exploratory behavior more generally, although a weakness is that the experimental design isn't directly studying exploration and instead studying the influence of these "off-task" variables on lever presses. Another weakness is the use of fiber photometry instead of other methods that allow cellular resolution measurements, since it is not clear what fiber photometry will reflect in such a heterogeneous population.

Specific comments

#	Reviewer comment	Editorial comment
1	Remarks to the Author: Strength of the claims Major comments:<br><br>1. A concern I had when reading the paper is what exactly is meant by “subjective experience” didn’t come into clear focus for me, at least while reading the intro. While I eventually understood what they were studying, I don’t think that the “experiences” under question (e.g. previous lever press duration, or magazine entries) are particularly subjective (at least any more so than the behavioral variable they are predicting, which is current trial lever press duration). Perhaps the sense they are subjective is that it is not necessarily adaptive that those variables correlate w/ the press duration (although not clear to me why use the term “subjective”). I realize that those behavioral influences on their actions are not overtly trained by the experimenter. As the authors point out, that may be why M2 lesions (which may reduce exploration based on previous work) reduces the influence of these “off-task” variables on press duration -- they may be more “exploratory” than “habitual”, and M2 (& nearby ACC) has been implicated in exploration. This is all to say that I had a lot of trouble understanding the significance of these behavioral variables that influenced lever press duration, and still do not fully grasp why M2 lesions affected the “off-task” variables only.	This point would need to be addressed for further consideration at Nature Communications.
2	2. I have concerns with using fiber photometry to record neural activity in a cortical population, which is highly heterogeneous. Not clear how the recorded signal would relate to the heterogeneous population activity.	This point would need to be addressed for further consideration at Nature Communications.

**3**	3. I found the main point of the big model of Supp Fig 2 unclear. It would really help this reader if the authors distilled what the take-aways were more clearly from all the model comparisons in the paper.	
**4**	4. In general, I found some of the conclusions from correlation data a bit too strong, as they suggested causality. E.g. “Thus checking behavior was a source of experiential information and influenced the subsequent executed behavior”. Wording seems too strong since correlation does not imply causality.	
**5**	5. I found their negative result from the reinforcement modeling to be a bit puzzling. Clearly the animals are using reinforcement to learn the task. So are they not modeling the effect of reward properly? If they stopped providing reward, surely the mice would stop doing the task.	
**6**	Minor:<br>1. I think the wording should be corrected when they refer to neurons “using experience” and “planning” - I think that’s something an animal but not a neuron can do. E.g. “M2-DMS projections use recent experience to plan upcoming actions”	
**7**	Remarks to the Author: Reproducibility I thought their reporting of the statistical tests & results was appropriate and in fact clearer than other papers I have seen published in these journals.	

**Reviewer #2**	
**Reviewer #2**	This reviewer has not chosen to waive anonymity. The reviewer’s identity can only be shared with representatives of an established journal editorial office.
**Reviewer #2 expertise**<br>Summarised by the editor	Decision making, learning, prefrontal circuits, in vivo imaging/recordings
**Editor’s comments about this**	The reviewer finds the paper to be of interest and provides an overall positive assessment, but has raised various concerns regarding the conceptual advance provided, and the scope of the data support for the conclusions. Please see major

**review**	comments.
**Reviewer #2 comments**	
**Overview**	Remarks to the Author: Overall significance<br><br>Schreiner et al. investigated how recent experiences (i.e. the history of various actions and outcomes, as well as time between actions and time in the session) influence the lever press behavior of mice, and the associated neural circuits. In order to study uninstructed self-paced and self-generated actions, the authors used a task where mice must press and hold down a lever for a minimum duration before they can earn a food reward upon lever release. The authors performed recordings and manipulations of the secondary motor cortex (M2) and its projections into the dorsal medial striatum (DMS) during the task. The main conclusions of the paper are that time and recent experience influence subsequent executed behavior and that M2 and M2-DMS projections convey information about recent experience to inform action execution.<br><br>The authors used a simple task and performed a wide range of techniques (projection-specific lesions, fiber photometry, closed-loop optogenetics inhibitions, and modeling). Each experiment includes a good number of animals and the data presented is carefully analyzed. In particular, I really appreciated that the authors rigorously characterized the behavior of mice. Overall, most of the results are convincing, but some need clarification.<br><br>However, I did not get a clear picture of the contribution of the paper. I applaud the author's view to de-roboticize rodents, but found the setup of the paper to be overly broad and therefore both not convincing and sometimes even confusing. Moreover, the neural data which constitute much of the study were not placed in a context where it was clear what insights were gotten. I encourage the authors to take a more focussed approach to the specific contributions of their study so that its interpretation could be more clear.

Specific comments

#	Reviewer comment	Editorial comment
1	Remarks to the Author: Strength of the claims Major points<br><br>1. The introduction and set-up of the paper fail in my opinion to set the appropriate context for the work presented. "Subjective experience" is used in philosophy and in the neuroscience of consciousness to refer to subjective phenomena experienced from the first person perspective and inaccessible to third-person (e.g. behavioral or physiological) measurements. It is the "what it is like to be" an individual organism (Nagel, 1974). This work does not	

	attempt to address this issue, so using the term adds unnecessary confusion. “Experience” is used in neuroscience to mean “dependent on the history of stimuli, inputs, events, etc”. For an individual animal, its experience is the history of events that happen to it, so an animal’s experience is necessarily “subjective”, and adding “subjective” to “experience” does not add anything: there can be no other kind of behavioral experience.	
**2**	2. The authors imply from the title that mice are being treated as automatons. Yet while I am disposed to agree with this critique, it is also true that there are a great many studies of behavior in rodents involving internal states such as hunger, or cognitive functions like memory which meet the authors definition of subjective experience. Moreover, even if it is true that decision-making studies usually focus on a narrow set of task variables, in recent years, contrary to the author’s claim (line 60 and line 243), there are a variety of studies that go beyond stimulus-response characterization, but also consider how past rewards, actions, and stimuli influence subsequent perceptual choices. In fact there exist many reports (including some cited in this paper like refs. 7 or 17, but also Berditchevskaia et al. 2016 or Ashwood et al. 2021 for instance) investigating how internal states, contextual information, and task-event history influence behavior. Also relevantly, there is a large literature on studies of priors and their influence on behavior over the last couple decades.	
**3**	3. The authors present the lever-press task as a relatively novel framework where “experience is essential and not merely incidental to decision making” (line 108). It was not clear what the authors had in mind here. Any task involving probabilistic rewards, waiting, foraging, exploration or reversals, which includes quite a large literature, also requires history-dependent decision-making. So the authors should try to zoom in more to get at what is special here.	
**4**	4. The authors emphasize the need to focus on individualistic behavior to better understand adaptive decision making. Although I agree with this perspective, the analyses performed in the manuscript actually fall short of what might be expected in terms of individual mouse level analyses.<br><br>The data presented here are all averages across sessions and mice. For instance, it is unclear whether different mice show different patterns of learning (Fig 1.) or whether the M2 activity is consistent across animals (Fig. 4). Is the behavior consistent across different	This point would be necessary to address for further consideration at Nature Communications.

	mice? If this is not the case, it would be interesting to know whether different use of prior experience can explain the variability observed across individuals.<br><br>Similarly, the design of the LME model does not allow different mice to treat prior experience differently. Yet, this seems important if the goal is to study subjective behavior. I acknowledge that it may not be possible to run individual LME models (due to a restricted amount of observations for each session or mouse). However, in theory, it should be possible to add a random term for each different predictors (not just for day and mouse) and check whether the beta coefficients differ across individuals.	
**5**	3. Comparing coefficients of different models can be prone to misinterpretations. Here, I could not find enough information (neither in the text nor in the methods) to judge whether the comparisons of the beta coefficients from the different models are to be fully trusted.<br><br>Fig. 2B and Fig. 3E: it seems that the magnitude of the coefficients of control mice in Fig. 2B is more similar to the coefficients of lesions rather than the sham (Fig. 3E). Although this is quite odd, the authors did not comment on this result. Also, are the r-squares of the three models similar? Were the predictors in the LME z-scored? The latter is quite important in order to compare the value of the coefficients.<br><br>From Table S4, it seems that the intercepts of Sham and Lesion are different. Yet in Fig. 3D, press duration in the two conditions is similar. Isn't that inconsistent? It would help to see a direct comparison of the coefficient with the mice from Fig. 2 (also for Fig. 3G), to have a better sense of the intrinsic variability of the coefficients. This comment also applies to Fig. 6.	
**6**	4. In Fig. 1, it is explained that for short level press criteria (800 ms), mice seem to mainly rely on estimated timing, while for long criteria (1600 ms) mice also rely on prior experience (as demonstrated in Fig. 2). If this is real, this result could be further exploited to strengthen the main conclusions.<br><br>It would be interesting to see the LME model applied to sessions with 800 ms criteria. In theory, for those sessions the coefficients of the previous trials should not be significant (nor the coefficients related to head entry, reward,or time), indicating that prior experience has no effect on lever press-duration in these sessions.<br><br>Similarly, inhibition of M2-DLM projections (or M2 lesions) during	This point would be necessary to address for further consideration at Nature Communications.

	the 800 ms session should not have such a dramatic effect as in the 1600 ms session.	
**7**	Minor points<br>1. What is Fig 4B reporting? The average Ca2+ activity of a single session, of several sessions of the same mouse, of all sessions of all mice? It would be nice to see that this result generalizes across recordings or at least across animals.<br>2. For model comparison of Ca2+ activity, do the authors also use the BIC?<br>3. An illustration of the different models (along with the Tables) would help.	

**Reviewer #3**	
**Reviewer #3**	This reviewer has not chosen to waive anonymity. The reviewer's identity can only be shared with representatives of an established journal editorial office.
**Reviewer #3 expertise**<br>Summarised by the editor	Action selection, learning, cortico-basal ganglia circuits, in vivo imaging/recordings
**Editor's comments about this review**	The reviewer finds the paper to be of interest, but has raised concerns regarding the conceptual advance provided, and the scope of the data support for the conclusions. Please see major comments.
**Reviewer #3 comments**	
**Overview**	Remarks to the Author: Overall significance<br>This paper examined the corticostriatal pathway from secondary motor cortex (M2) and dorsomedial striatum (DMS). They hypothesize that M2 projections to the DMS integrate experiential information to guide exploration-based decision-making. The authors suggest that the role of "subjective experience", defined as internal, experiential, temporal, and contextual information, is neglected in traditional decision-making tasks because of the reliance on trial-based designs, which ignore continuous and self-initiated behavior that may underlie changes in neural activity. To test their hypothesis, they combine lesions, population level calcium recordings, and optogenetic manipulation, with self-generated lever press hold task to

	determine a role for M2-DMS neurons in decision making. It was argued that different aspects of subjective experience is represented in this pathway and used for action planning.<br>The authors performed some careful analysis of behavior in their study, and the results are interesting. But there are major flaws with their arguments and the main conclusions are not strongly supported by the data.<br>Remarks to the Author: Impact<br>Neuropsychopharmacology would be suitable

Specific comments

#	Reviewer comment	Editorial comment
1	Remarks to the Author: Strength of the claims<br>1. Introduction- A primary assumption of the paper is that current trial based methods of investigation do not account for the ‘subjective experience’. However, the authors define ‘subjective experience’ as “internal, experiential, temporal, and contextual information”. This appears to include just about everything. With this definition, it would be impossible to design an experiment where the subject can’t access ‘subjective experience’. I don’t see how a claim like this can ever be falsified. In general, I suggest the authors provide a more precise definition of ‘subjective experience’.	
2	2. The authors argue that neuroscientists have ignored subjective experience because they only focus on a few task-relevant variables when studying mouse behavior. This is misleading. While it could be argued that mouse behavioral tasks lack ethological validity or are too simplistic for understanding higher functions, it is not accurate to say that the variables typically studied have nothing to do with subjective experience. Concepts like decision variable, value, utility, uncertainty, risk, etc. seem to be related to subjective experience, and few would deny that. In many cases the designs are based on previous monkey studies, so does that mean that monkey researchers also make the implicit assumption that monkeys are automatons without any subjective experience? The authors, in my opinion, are mostly attacking a straw man here. It is also unclear exactly which aspects of current designs are inadequate: that they are cued, that they have trials, that they are binary? If so, it is not explained why these features are problematic. I think what the authors are really trying to say is that previous work on decision making focus on a narrow set of variables and use behavioral task	This point would be necessary to address for further consideration at Nature Communications.

	designs that may not reveal the underlying mechanisms, not that they ignore subjective experience, or that mice are assumed to be automatons in previous studies.	
**3**	3. The data in Figures 1 do not support the author's conclusion that the animal does not solely rely on timing the press duration. Because the IQR does not similarly increase with the median across training or within the last session, the authors conclude the behavior violates the scalar property. There is however no direct test for the scalar property: statistical comparison of the IQR/median ratio once the animal has fully learned the task. In addition, whether timing is scalar or whether they use timing information on this task are separate issues. Violation of scalar property has been reported before, but that doesn't mean the animal isn't timing. What the authors have demonstrated is systematic variability within session that is not clear from traditional summary statistics. But it's misleading to equate timing with scalar property.	This point would be necessary to address for further consideration at Nature Communications.
**4**	4. The authors conclude that, due to the significant main effects of the 2-way RM ANOVA in Figure 1I, consecutive presses that were 2 standard deviations above the mean did not occur by chance. The first day of training is confounded by learning. The trained data should be compared in this analysis. Because the 1st day of training for each duration requirement in the analysis appears to be driving the main effect, the data likely does not support the authors claim that "recent experience may contribute to adaptive behavior".	
**5**	5. The finding that M2 lesioned mice were inflexible and failed to incorporate a number of variables that normal mice use is interesting, but not that novel given previous work. This could presumably be explained by some type of working memory deficit for recent actions.	
**6**	6. Photometry data only show population activity. It's not surprising that such activity would represent different behavioral variables. Is there a brain region where this is not true?	
**7**	7. The template/pattern matching hypothesis is interesting, but I don't see how it is supported by the optogenetic data, which can be explained by failure to use working memory or efference copy of recent actions.	
**8**	Minor points<br><br>Line 144: missing words after 'variability'	

	Line 608-the laser power for optogenetic experiments should be reported<br>Line 92-M2 is not itself a "circuit" but a brain region<br>Line 290-missing an "is" after M2 activity<br>Conclusion- there should be some discussion of whether prior work supports that the secondary motor cortex could 'integrate' the variables discussed (e.g. head checks, prior lever presses, Time, etc.). How does such information reach M2?	
**9**	Remarks to the Author: Reproducibility Analysis seems appropriate.	

Open research evaluation

Data availability

Data availability statement

Please add a Data Availability statement. Please ensure that your Data Availability statement includes accession details for deposited data, mentions where Source data can be found, and states that all other data are available from the corresponding author (or other sources, as applicable) on reasonable request. More information about our data availability policy can be found here: https://www.nature.com/nature-portfolio/editorial-policies/reporting-standards#availability-of-data See here for more information about formatting your Data Availability Statement: http://www.springernature.com/gp/authors/research-data-policy/data-availabilitystatements/12330880

Thank you for including a Data Availability statement. However, we noted that you have only indicated that data are available upon request. The data availability statement must make the conditions of access to the "minimum dataset" that are necessary to interpret, verify and extend the research in the article, transparent to readers. In addition, Nature Portfolio policies include a strong preference for research data to be archived in public repositories. For data types without specific repositories, we recommend that data are deposited in a generalist repository such as figshare or Dryad. More information about our data availability policy can be found here: https://www.nature.com/nature-portfolio/editorialpolicies/reporting-standards#availability-of-data See here for more information about formatting your Data Availability Statement: http://www.springernature.com/gp/authors/research-data-policy/data-availabilitystatements/12330880

Other data requests

All source data underlying the graphs and charts presented in the main figures should be made available as Supplementary Data (in Excel or text format) or via a generalist repository (eg, Figshare or Dryad). This is strongly encouraged for publication in a Nature Portfolio journal, but is also best practice for publication in any venue.

Please ensure that a Source Data file is included with your resubmission. The Source Data file contains the raw data underlying the following types of display items:

- Any reported means/averages in box plots, bar charts, and tables
- Dot plots/scatter plots, especially when there are overlapping points
- Line graphs

The data should be provided in a single Excel file with data for each figure/table in a separate sheet, or in multiple labelled files within a zipped folder.

The file should be labelled 'Source Data' and should be mentioned in all relevant figure legends using the template text below:

"Source data are provided as a Source Data file.". The “Data Availability” section should also include the statement “Source data are provided with this paper.”

To learn more about our motivation behind this policy, please see:

https://www.nature.com/articles/s41467-018-06012-8. An example of the Source Data file is available demonstrating the correct format:

https://www.nature.com/documents/ncomms-example-source-data.xlsx

Code availability and citation

Please include a statement under the heading "Code Availability", indicating whether and how the custom code/software reported in your study can be accessed, including any restrictions to access. This section should also include information on the versions of any software used, if relevant, and any specific variables or parameters used to generate, test, or process the current dataset. Code availability statements should be provided as a separate section after the Data Availability section. Upon publication, Nature Portfolio journals consider it best practice to release custom computer code in a way that allows readers to repeat the published results. Code should be deposited in a DOI-minting repository such as Zenodo, Gigantum or Code Ocean and cited in the reference list following the guidelines described in our policy pages (see link below). Authors are encouraged to manage subsequent code versions and to use a license approved by the open source initiative. Full details about how the code can be accessed and any restrictions must be described in the Code Availability statement.

See here for more information about our code availability policies:
https://www.nature.com/natureportfolio/editorial-policies/reporting-standards#availability-of-computer-code

We also provide a Code and Software submission checklist that you may find useful:
https://www.nature.com/documents/nr-software-policy.pdf

Please note: because of advanced features used in this form, you must use Adobe Reader to open the documents and fill it out.

Ethics

Please provide a 'Competing interests' statement using one of the following standard sentences: 1. The authors declare the following competing interests: [specify competing interests] 2. The authors declare no competing interests. See our competing interests policy for further information:

https://www.nature.com/natureresearch/editorial-policies/competing-interests

Because your study uses live vertebrates, a statement affirming that you have complied with all relevant ethical regulations for animal testing and research is necessary. A statement explicitly confirming if the study received ethical approval, including the name of the board and institution that approved the study protocol is also required. The species, strain, sex and age of animals should be included.

Reporting and reproducibility

Reporting

For all statistics (including error bars), please provide the EXACT n values used to calculate the statistics (reporting individual values rather than a range if n varied among experiments) AND define type of replicates (e.g., cell cultures, technical replicates). Please avoid use of the ambiguous term “biological replicates”; instead state what constituted the replicates (e.g., cell cultures, independent experiments, etc.). For all representative results, indicate number of times experiments were repeated, number of images collected, etc. Indicate statistical tests used, whether the test was one or two-tailed, exact values for both significant and non-significant P values where relevant, F values and degrees of freedom for all ANOVAs and t-values and degrees of freedom for t-tests. **If this is too much information to include in the figure legends, we recommend providing it as a supplementary table and referencing the table in the statistics section.**

Reproducibility

A Word document indicating revisions that need to be made in compliance with our reporting summary is attached. The detailed comments document lists all of the changes that need to be made to the text, and particularly the main and supplementary figure legends, including (but not limited to) details regarding sample sizes, replication, scale and error bars, and statistics. Please include a statement indicating how the sample sizes were chosen in the Methods section. If a power analysis was used, provide the details of this analysis. If you did not use a power analysis, the following is sufficient: "No statistical methods were used to pre-determine sample sizes but our sample sizes are similar to those reported in previous publications (ref x,y,z)." Stating that sample sizes were chosen to demonstrate statistically significant effects is only accurate if you performed an a priori test to determine this value. Please indicate whether the data met the assumptions of the statistical tests used, including whether normality and equal variances were formally tested. If not, please show data distribution (individual data points) and include the following statement: "Data distribution was assumed to be normal but this was not formally tested.” Please include a statement on randomization in the Methods. Indicate whether the data collection was randomized or appropriately blocked, how animal/samples were assigned to the various experimental groups and whether there was any randomization in the organization of the experimental conditions or stimulus presentations. Please include a statement indicating whether blinding was used in the Methods. If

there was no blinding, this must be clearly stated in the manuscript, as follows: "Data collection and analysis were not performed blind to the conditions of the experiments." Please disclose whether any animals or data points were excluded from the analyses for any reason and note the rationale for the exclusions.

Statistics

Error bars should be displayed wherever possible and must be clearly defined in the caption for each figure. To improve reproducibility of your analyses, please provide details regarding your treatment of outliers.

Methods descriptions

The Methods must contain sufficient detail such that the work could be replicated. It is preferable that all key methods be included in the main manuscript, rather than in the Supplementary Information.

The methods section can be around 3,000 words in length (no strict limit), they can contain references that do not count towards the reference limit in the main paper, and will be fully indexed. You should feel free, and we in fact encourage you, to incorporate any part of your Supplementary Information that you feel is important for the rest of the paper within this section.

The Methods section should be written as concisely as possible but should contain all elements necessary to allow interpretation and reproduction of the results (please note, however, that the methods section cannot contain any figures or tables at present).

If there are additional references in the Methods section, their numbering should continue from the last reference in the main paper, and the list should follow the Methods section.

Other notes

We have included as an attachment to the decision letter a version of your Reporting Summary with a few notes. This is mainly for your information, but we hope it is helpful when preparing a revised manuscript. If you decide to resubmit the manuscript for further consideration, please be sure to include an updated Reporting Summary.
