# Supplementary material for: Information normally considered task-irrelevant drives decision-making and affects premotor circuit recruitment
Source: Nat Commun. 2022 Apr 19;13:2134. doi: 10.1038/s41467-022-29807-2 (PMC9018678; doi:10.1038/s41467-022-29807-2)
Supplement: Supplementary file 3 — Reporting Summary [file 41467_2022_29807_MOESM3_ESM.pdf]

nature portfolio

Corresponding author(s): Christina Gremel

Last updated by author(s): Feb 15, 2022

Reporting Summary

Nature Portfolio wishes to improve the reproducibility of the work that we publish. This form provides structure for consistency and transparency in reporting. For further information on Nature Portfolio policies, see our Editorial Policies and the Editorial Policy Checklist.

Statistics

For all statistical analyses, confirm that the following items are present in the figure legend, table legend, main text, or Methods section.

n/a	Confirmed	
☐	☒	The exact sample size (*n*) for each experimental group/condition, given as a discrete number and unit of measurement
☐	☒	A statement on whether measurements were taken from distinct samples or whether the same sample was measured repeatedly
☐	☒	The statistical test(s) used AND whether they are one- or two-sided<br>*Only common tests should be described solely by name; describe more complex techniques in the Methods section.*
☐	☒	A description of all covariates tested
☐	☒	A description of any assumptions or corrections, such as tests of normality and adjustment for multiple comparisons
☐	☒	A full description of the statistical parameters including central tendency (e.g. means) or other basic estimates (e.g. regression coefficient) AND variation (e.g. standard deviation) or associated estimates of uncertainty (e.g. confidence intervals)
☐	☒	For null hypothesis testing, the test statistic (e.g. *F*, *t*, *r*) with confidence intervals, effect sizes, degrees of freedom and *P* value noted<br>*Give P values as exact values whenever suitable.*
☒	☐	For Bayesian analysis, information on the choice of priors and Markov chain Monte Carlo settings
☒	☐	For hierarchical and complex designs, identification of the appropriate level for tests and full reporting of outcomes
☒	☐	Estimates of effect sizes (e.g. Cohen's *d*, Pearson's *r*), indicating how they were calculated

Our web collection on statistics for biologists contains articles on many of the points above.

Software and code

Policy information about availability of computer code

Data collection	Med-PC operant chambers and software were used to timestamp and collect data (Med-Associates IV). Arduino Unos (Arduino) and Bonsai software (Bonsai 2.3.1) were used to control light delivery and GCaMP recording experiments.
Data analysis	Matlab2019b (Mathworks), Excel (Microsoft) RM correlation package (R Core team), and Prism 6 (Graphpad) were used for analysis. Custom Matlab code is available at http://github.com/gremellab/Hold-Down-Behavior-GCAMP-Opto-analysis

For manuscripts utilizing custom algorithms or software that are central to the research but not yet described in published literature, software must be made available to editors and reviewers. We strongly encourage code deposition in a community repository (e.g. GitHub). See the Nature Portfolio guidelines for submitting code & software for further information.

Data

Policy information about availability of data

All manuscripts must include a data availability statement. This statement should provide the following information, where applicable:

- Accession codes, unique identifiers, or web links for publicly available datasets
- A description of any restrictions on data availability
- For clinical datasets or third party data, please ensure that the statement adheres to our policy

All data supporting the findings of this study are provided within the paper and its supplementary information. A source data file is provided with this paper. All additional information will be made available upon reasonable request to the authors.

Field-specific reporting

Please select the one below that is the best fit for your research. If you are not sure, read the appropriate sections before making your selection.

☒ Life sciences ☐ Behavioural & social sciences ☐ Ecological, evolutionary & environmental sciences

For a reference copy of the document with all sections, see nature.com/documents/nr-reporting-summary-flat.pdf

Life sciences study design

All studies must disclose on these points even when the disclosure is negative.

Sample size	The mouse sample sized were selected based on pilot experiments to determine behavioral effect size, and prior works published in this brain area using a similar task.
Data exclusions	Mice without sufficient viral expression or with misplaced optical ferrules (determined via microscope) were excluded from analysis. Additionally, mice who failed to acquire lever pressing behavior during the pretraining phase were also excluded from analysis.
Replication	Experimental manipulations during behavior were performed across 2-3 replicates of 8 and were similar across all experiments. We used the same linear mixed effect model analysis on all data sets, allowing us to see that our results were replicable across control groups in all experiments (all exceptions noted in discussion).
Randomization	Animals were randomly selected for experimental or control groups within individual experiments. We counterbalanced animal sex, cage, age, and the operant box in which they underwent training.
Blinding	Investigators were blinded to experimental condition during data collection and histology. Analysis using models was done with group comparisons, hence animals were identified on a group basis.

Reporting for specific materials, systems and methods

We require information from authors about some types of materials, experimental systems and methods used in many studies. Here, indicate whether each material, system or method listed is relevant to your study. If you are not sure if a list item applies to your research, read the appropriate section before selecting a response.

Materials & experimental systems

n/a	Involved in the study	
☒	☐	Antibodies
☒	☐	Eukaryotic cell lines
☒	☐	Palaeontology and archaeology
☐	☒	Animals and other organisms
☒	☐	Human research participants
☒	☐	Clinical data
☒	☐	Dual use research of concern

Methods

n/a	Involved in the study	
☒	☐	ChIP-seq
☒	☐	Flow cytometry
☒	☐	MRI-based neuroimaging

Animals and other organisms

Policy information about studies involving animals; ARRIVE guidelines recommended for reporting animal research

Laboratory animals	Similar numbers of male and female C57Bl/6J mice (>7 week/50PND) (The Jackson Laboratory, Bar Harbour, ME) were used for all experiments. Animal housing was maintained at 71.6 degrees F with 30% humidity.
Wild animals	This experiment did not involve wild animals.
Field-collected samples	The study did not involve field collected samples.
Ethics oversight	All experiments were approved by the University of California San Diego Institutional Animal Care and Use Committee and were carried out in accordance with the National Institutes of Health (NIH) "Principles of Laboratory Care".

Note that full information on the approval of the study protocol must also be provided in the manuscript.
